# Identification and Functional Expression of a Glutamate- and Avermectin-Gated Chloride Channel from *Caligus rogercresseyi*, a Southern Hemisphere Sea Louse Affecting Farmed Fish

**DOI:** 10.1371/journal.ppat.1004402

**Published:** 2014-09-25

**Authors:** Isabel Cornejo, Olga Andrini, María Isabel Niemeyer, Vanessa Marabolí, F. Danilo González-Nilo, Jacques Teulon, Francisco V. Sepúlveda, L. Pablo Cid

**Affiliations:** 1 Centro de Estudios Científicos (CECs), Valdivia, Chile; 2 UPMC Université Paris 06, UMR_S 1138, Team 3, Paris, France; 3 INSERM, UMR_S 872, Paris, France; 4 Universidad Andrés Bello, Centro de Bioinformática y Biología Integrativa, Facultad de Ciencias Biológicas, Santiago, Chile; Copenhagen University, Denmark

## Abstract

Parasitic sea lice represent a major sanitary threat to marine salmonid aquaculture, an industry accounting for 7% of world fish production. *Caligus rogercresseyi* is the principal sea louse species infesting farmed salmon and trout in the southern hemisphere. Most effective control of *Caligus* has been obtained with macrocyclic lactones (MLs) ivermectin and emamectin. These drugs target glutamate-gated chloride channels (GluCl) and act as irreversible non-competitive agonists causing neuronal inhibition, paralysis and death of the parasite. Here we report the cloning of a full-length CrGluClα receptor from *Caligus rogercresseyi*. Expression in *Xenopus* oocytes and electrophysiological assays show that CrGluClα is activated by glutamate and mediates chloride currents blocked by the ligand-gated anion channel inhibitor picrotoxin. Both ivermectin and emamectin activate CrGluClα in the absence of glutamate. The effects are irreversible and occur with an EC_50_ value of around 200 nM, being cooperative (n_H_ = 2) for ivermectin but not for emamectin. Using the three-dimensional structure of a GluClα from *Caenorabditis elegans*, the only available for any eukaryotic ligand-gated anion channel, we have constructed a homology model for CrGluClα. Docking and molecular dynamics calculations reveal the way in which ivermectin and emamectin interact with CrGluClα. Both drugs intercalate between transmembrane domains M1 and M3 of neighbouring subunits of a pentameric structure. The structure displays three H-bonds involved in this interaction, but despite similarity in structure only of two these are conserved from the *C. elegans* crystal binding site. Our data strongly suggest that CrGluClα is an important target for avermectins used in the treatment of sea louse infestation in farmed salmonids and open the way for ascertaining a possible mechanism of increasing resistance to MLs in aquaculture industry. Molecular modeling could help in the design of new, more efficient drugs whilst functional expression of the receptor allows a first stage of testing of their efficacy.

## Author Summary

Sea lice are the main parasites affecting farmed salmon and trout in the world. *Caligus rogercresseyi* is the principal sea louse species infesting farmed fish in the southern hemisphere. Successful control of these parasites has been achieved using macrocyclic lactones (MLs), but resistance has emerged over time. In other invertebrates, MLs target membrane receptors regulating synaptic transmission in the parasite nervous system. Here we identify and study the function of such a receptor from *Caligus rogercresseyi*, and gain an idea about how two MLs, ivermectin and emamectin, interact with the receptor to produce their effects. Our molecular modeling of the protein in complex with the drugs suggests a novel way in which ivermectin and emamectin exert their effects on CrGluCl due to a lack of conservation at interaction sites identified in the crystal structure of the receptor from *C. elegans*. We believe that the identification of a ML target in sea louse will aid the study of drug-resistance mechanisms and could help in the design of new, more efficient antiparasitic drugs.

## Introduction

Sea lice are marine ectoparasite copepods of the Caligidae family (order Siphonostomatoida) that attach to host marine fish and feed on their epidermal tissue, and blood. The Caligidae family is constituted by hundreds of species belonging to the *Caligus* and *Lepeoptherius* genera. Increasing interest in these parasites has arisen owing to the ravages they produce on fish aquaculture leading to morbidity and mortality with extremely high economic impact in the industry. *Lepeoptherius salmonis* is the most important caligid species affecting Northern hemisphere salmon and trout aquaculture, and its biology, sensitivity to chemotherapeutic drugs and distribution have been and presently are very actively studied [1]. A different sea louse, *Caligus rogercresseyi* is the most important parasite affecting Atlantic salmon and rainbow trout sea water farming in Chile. This *Caligus* was described as a separate species only in the year 2000 [2] and much remains to be known about its biology. As in the Northern hemisphere, *Caligus rogercresseyi* infestation within the nationally important Chilean aquaculture industry is associated with increased costs and decreased productivity with high social impact [Alvial et al. cited in 3].

Chemical treatment has been used in fish farms to combat sea lice infestation with variable degree of success. Compounds used include organophosphates, hydrogen peroxide, pyrethroids, chitine synthesis inhibitors and avermectins [3]. Emamectin and ivermectin are macrocyclic lactone avermectins that have been widely used to control parasitic infections in humans and animals. Emamectin benzoate has been the treatment of choice for sea lice treatment given its efficacy and ease of administration. The compound, which is formulated as SLICE (Merck Animal Health), is administered orally with fish feed and provides long-lasting protection against all forms of attached sea lice.

Studies in nematodes have shown that avermectins interfere with synaptic transmission through irreversible activation of glutamate-gated chloride channel receptors leading to eventual paralysis and death of the parasites [4]. Though highly successful as antiparasitic drugs over extended periods of time, resistance to avermectins has emerged and has become a major problem worldwide [5].

Glutamate-gated chloride channels (GluCls) belong to the Cys-loop receptor, or also known as pentameric ligand gated ion channel, family which is widely present in nematodes and insects [6]–[10]. Structurally they are pentamers and can assemble as homopentamers or heteropentamers. Functionally characterized α and β subunits (GluCl-α and GluCl-β subunits), also named GLC-1 and GLC-2, have been reported in the nematodes *C. elegans* [11], [12] and *Hemonchus contortus* [13]. The *C. elegans* GluCl family now extends to six genes: *glc-1* to *glc-4*, and *avr-14* and *avr-15* [14]. In *Drosophila melanogaster* only one subunit is present, DmGluClα, which is responsible for the insect sensitivity to avermectin compounds [15]. The same seems to be the case in most insects studied [14]. Recently our understanding of the structural and functional properties of these receptors has made an enormous progress thanks to development of the crystallographic structure of *Caenorabditis elegans* GluClα subunit (CeGluClα) [16]. The structure revealed details of the agonist binding site in the extracellular domain as well as the ivermectin site. The physiological role of these receptors in invertebrates is to mediate and regulate the inhibitory synapses and cellular excitability making them a very important and effective pharmacological target for insecticides [14].

Emamectin has been used to combat infestation by *Caligus rogercresseyi* in Chilean farmed salmon systems since 2000 [2], but reports of growing resistance to the drug have emerged [17]. A detailed knowledge of the molecular mechanisms of this resistance is essential in devising strategies to circumvent it. We think that characterizing the pharmacological target of emamectin benzoate from *C. rogercresseyi* could help to understand the resistance mechanisms and to design new drugs. Given that this molecular entity is not known in this species, our aim in the present work was to identify an emamectin sensitive chloride channel and characterize its electrophysiological properties in oocytes of *Xenopus laevis* and, taking advantage of the availability of a molecular structure of member of the pentameric ligand gated ion channel family of proteins, to obtain clues about the mode of action of the avermectins.

## Materials and Methods

### Sea louse specimens

*Caligus rogercresseyi* specimens were obtained from three different sources. The first were specimens obtained locally at the Universidad Austral, Valdivia, Chile. The product (see below) obtained from these specimens is hereafter referred to as CrGluCl-Vald. The other sources were salmonid cages located in the Darwin and Errázuriz Channels in Southern Chile, and their products are referred to as CrGluCl-Dw and CrGluCl-Err, respectively. All samples were collected *in situ* and immediately stored in RNA-later protective solution (Ambion) at −20°C until use. Pools of 6 to 10 adult specimens were pulverized under liquid nitrogen and immediately mixed with Trizol reagent (Invitrogen) to extract total RNA following the supplier instructions. The RNA concentration was quantified based on absorbance at 260 nm and its integrity evaluated by agarose gel electrophoresis.

### Cloning of the glutamate gated chloride channel

Two degenerate oligonucleotides were designed based on an amino acid region highly conserved in arthropod glutamate gated chloride channels (GenBank accession number: NP_001171232, NP_001071277, NP_001103244, NP_732447, ABI95855). The forward (PCRdegFor(d64)) and reverse (PCRdegRev(d16)) corresponds to MEYSVQLTFRE and KTNTGEYSC amino acid sequences respectively. The reverse transcription reaction was performed in 5 µg of total RNA primed with oligodT and random primers using Superscrit II kit (Invitrogen). KOD Hot Start DNA polymerase (Novagen) was used for PCR amplification and the amplicon was subcloned in pGEM-T (Promega) vector and analyzed by sequencing. The 5′ cDNA was obtained by 5′RACE and inverse PCR based on the protocol published by Huang, which combines the template-switching effect with inverse PCR [18]. Briefly, the reverse transcription conditions using Superscrit II were basically as supplier recommends except that the mix contained T-S primer for template switching effect. One µl of reverse transcription was used to synthesize the second strand with T-S PCR and PCRdegRev(d16) primers and KOD Hot Start DNA polymerase. The PCR product was purified by column (Wizard, Promega) and quantified. 200 ng of the product were phosphorylated with Polynucleotide kinase (New England Biolabs) and then ligated with T4 DNA ligase (Fermentas). That ligated product was used as template for inverse PCR (PCRi). A PCRi number 1 was performed with PCRiFor1 and PCRiRev1 primers and the product was used as template for PCRi number 2 using PCRiFor2 and PCRiRev2 primers. The 3′ cDNA end was obtained using oligodT with an anchor sequence (cDNA Cloning Primer) in the reverse transcription reaction. The second strand was synthesized with KOD Hot Start DNA Polymerase using 1 µl of transcription reverse product as template and PCRiFor1 and PCR RACE 3′ primers (first PCR). A second PCR reaction was performed using as template the PCR product from the first reaction with the PCRiFor2 and PCR RACE 3′ primers. To obtain the full length open reading frame (ORF) the cDNA was amplified with the PCRfullFor and PCRfullRev primers. The same cDNA was used to amplify 3 partial fragments by conventional PCR using the KOD Hot Start DNA polymerase and the primers PCRfullFor and PCRiRev1, PCRmidFor and PCRmidRev and PCR3′For and PCR 3′Rev. All the PCR products were purified, cloned in the pGEM-T Easy vector (Promega) and sequenced with T7 and SP6 primers. The full ORF was excised from this vector with NotI enzyme and subcloned in the pCR3.1 vector in the same site. The oligonucleotides used in the cloning procedure are listed in Table S1.

### Expression vector construction

To express the channels in *Xenopus laevis* oocytes, the ORF was cut with BglII and XbaI from crGluCl/pCR3.1 and directionally subcloned in the pTLB and a BamHI site was inserted downstream to the stop codon.

### cRNA preparation

The plasmids crGluCl/pTLB were linearized with BamHI enzyme (Roche) and used as template for synthesis of capped cRNA using mMessage Machine SP6 kit (Ambion, Austin, TX, USA).

### Voltage-clamp in *Xenopus laevis* oocytes

Defolliculated oocytes were injected with 5 ng, 10 ng and 20 ng of each cRNA and kept at 16°C in modified Barth's solution for 5 days. Two-electrode voltage clamp recording were performed at room temperature using TURBO TEC-10CX (npi electronic GmbH, Tamm, Germany) and PClamp 9 software (Axon Instruments, USA), from 2 to 4 days after oocyte injection. Oocytes were recorded in a chamber (Model RC-1Z, Warner Instrumenst, USA) under continuous superfusion. Electrodes were pulled to 0.5–2 MΩ and filled with 3 M KCl. The bath reference was 3 M KCl in a 3% agar bridge connected to an Ag–AgCl pellet. Currents were recorded in response to a ramp protocol consisting in a holding potential period of −30 mV for 40 ms followed by a voltage jump to −100 mV for 20 ms. The ramp was from −100 mV to +60 mV with a 360 ms duration. The data were filtered at 1 kHz, digitized using a digidata 1440A analogue-to-digital converter and analyzed using Axon pClamp 9 software. The solution bathing the oocytes during electrophysiological recordings had the following composition (mM): 115 NaCl, 2 KCl, 1.8 CaCl_2_, 1 MgCl_2_, 10 HEPES pH 7.5 obtained with NaOH. For the low [Cl^−^]_o_ solution all NaCl was replaced with the gluconate Na salt. Stock solutions of the drugs in DMSO were: emamectin 10 mM, ivermectin 10 mM, picrotoxin 100 mM. Different concentrations of the drugs were obtained by dilution in bathing solution. The highest DMSO concentration used was seen to have no effect on CrGluCl currents. Analysis of concentration-dependence of glutamate or drug effects was done by fitting a Hill model to the data using the non-linear regression suite of Sigmaplot 12.

### Molecular modelling of CrGluClα structure

In the absence of structural data for the GluCl receptor of *Caligus rogercresseyi* (CrGluClα), we built a model to help our understanding of experimental results. CrGluClα shares a 53% sequence identity with CeGluClα whose high resolution X-ray diffraction structure has been resolved [16] (comparison of similarly truncated CrGluClα as crystallized CeGluClα). We used these data (PDB ID 3RHW) as a reference structure to build a homology model of CrGluClα using Modeller v 9.10 software [19]. The molecular model was embedded into a 130×130 Å POPC lipid bilayer in a water box. The hydrated system was neutralized with NaCl at a concentration of 150 mM. The system was submitted to a molecular dynamics simulation under periodic bordering conditions (135×135×165 Å^3^) and isobaric-isothermal ensemble (NPT). The full system was relaxed through molecular dynamics (MD) simulations using NAMD 2.9 software [20] for 1 ns and subsequently equilibrated for 20 ns.

### Docking simulations

The homology model structure was used in docking simulations to identify ivermectin and emamectin binding sites in CrGluClα. The molecular docking procedure used Glide [21] software of the Schrödinger suite. The drugs, that belong to the macrocyclic lactone group, were designed using Maestro 2D sketcher [22], and prepared with LigPrep [23]. The CrGluClα model was prepared using the Protein Preparation Wizard [24] panel, assigning bond order, adding missing hydrogen atoms, correcting metal ionization states and creating disulphide bonds.

Grid size and position was defined from the position of ivermectin observed in the CeGluClα-ivermectin complex crystal structure. The box centre was defined as the midpoint of a line joining the two most distant atoms of the ligand core. The size of the grid was fixed at 22 Å, considering a distance of 34×34×34 Å in the coordinate axis.

A flexible docking was done using the OPLS-AA force field with standard precision (SP). This approach is appropriate for ligand screening in model-derived structures. To internally generate conformations, the ligand was considered flexible during the coupling process. A postdocking minimization allowed optimization of bond lengths and angles, including torsion angles. Owing the fact that this kind of docking code has not been adjusted to transmembrane conditions some spatial restraints were applied to guide the conformational sampling in the binding site. In order to try and reproduce the H-bonding network observed in the ivermectin binding in the crystallographic data [16], distance restraints were applied between Thr305 (Ser321 in the crystal) and ivermectin (and emamectin) O10 and between Leu263 and ivermectin (and emamectin) O13.

### Molecular dynamics

Three systems were considered: CrGluClα, and the CrGluClα-ivermectin and CrGluClα-emamectin complexes. Each system was embedded into a POPC lipid bilayer in a water box (dimensions: 135×135×165 Å^3^) and a NaCl concentration of 150 M. NAMD software [20] was used to relax each system through molecular dynamics (MD) simulations. MD was performed with the CHARMM force field and the TIP3P water model [25]. The temperature was kept at 300°K with the isobaric-isothermal ensemble using Langevin dynamics with a damping coefficient of 1 ps^−1^. By means of the Langevin Piston method the pressure was fixed at 1 atm. The equations of motion were integrated employing the Verlet r-RESPA algorithm [26] with a time step of 2 fs. All systems were subjected to energy minimization using periodic boundary conditions.

### Pore radius calculations

After a brief stabilization of each system, HOLE algorithm [27] was used to determine channel pore dimensions, by using the coordinates of the ion channel. Pore dimensions obtained considered the last 10 ns of trajectory (total time 100 ns).

## Results

### Cloning of the glutamate gated chloride channel

The GeneBank database was explored for the presence of sequences for glutamate-gated chloride channels belonging species phylogenetically close to *Caligus rogercresseyi*. The search produced sequences for *Lepeoptheirus salmonis*, *Drosophila melanogaster*, *Nasonia vitripennis*, *Apis mellifera* and *Tribolium castaneum*, which were used to design degenerated primers to attempt cloning a fragment of the receptor from *C. rogercresseyi* total RNA. The PCR amplification with the degenerated primers (Table S1) gave rise to a product of approximately 450 bp which included the two extracellular domain Cys-loops characteristic of these receptors. The predicted amino acid sequence of this fragment showed a 90% identity with the sequence from *L. salmonis* and 74% with that of *D. melanogaster.* Based on this sequence we designed specific primers to amplify the cDNA ends of the channel. In the 5′ and 3′ RACE experiments we obtained PCR products of 700 bp (5′) and 900 bp (3′). The predicted amino acid sequences were 79% (5′) and 83% (3′) identical to those of a putative receptor from *L. salmonis* and 62% (5′) and 55% (3′) with that of *D. melanogaster*. In the 5′ RACE sequence we found an ATG with many upstream in frame stop codons. We considered this ATG as the start codon.

**Figure 1:**
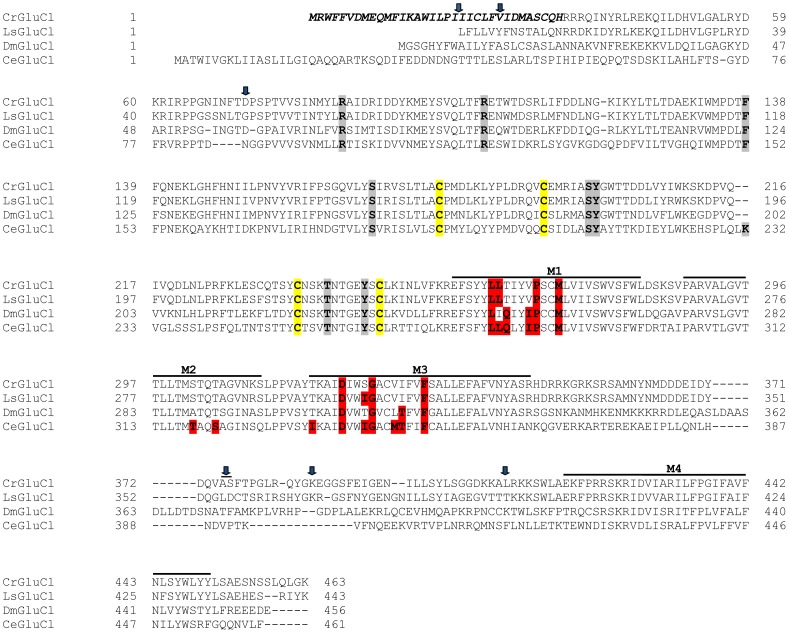
Alignment of CrGluClα amino acid sequence with other glutamate-gated chloride channel α subunits. PROMALS3D program (http://prodata.swmed.edu/promals3d/) was used to generate an alignment with the following glutamate receptor GluClα proteins: *L. salmonis* (Ls) GI:115361509, *D. melanogaster* (Dm) GI:1507685 and *C. elegans* (Ce) GI:559559. The predicted signal peptide of CrGluClα, identified using SignalP 4.1 programme, is shown in bold italic. M1–M4 are transmembrane domains; cysteines involved in C-loops are highlighted in yellow; in grey and in red are residues involved in glutamate and ivermectin binding respectively. Arrows indicate amino acids variations between *Caligus* obtained from different sources (see Figure 2). The position of the transmembrane domains and binding sites is based on the crystal structure of the *C. elegans* GluClα [16].

**Figure 2:**
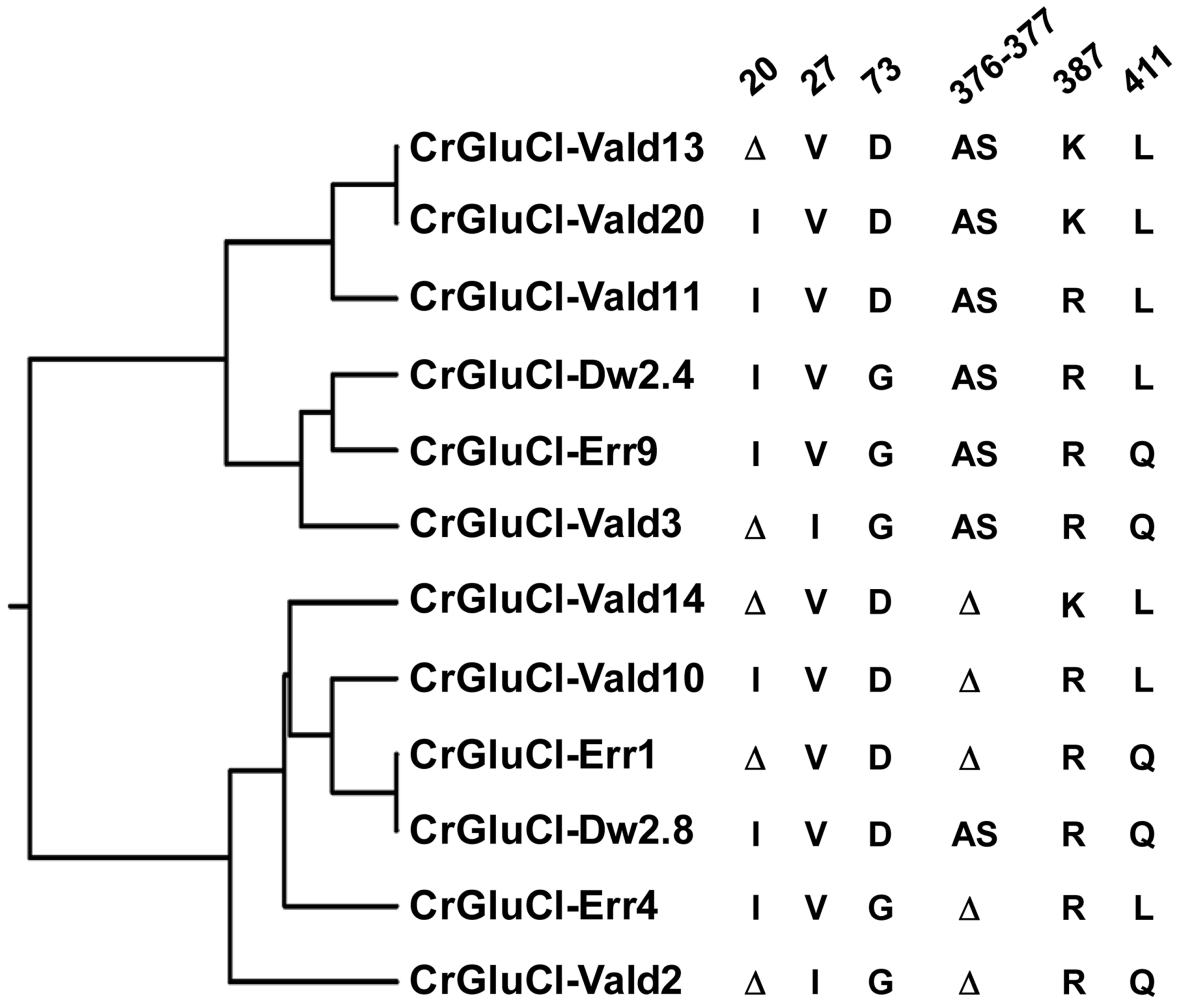
Variations in deduced CrGluClα primary structure. The rooted phylogenetic tree was done by using the UPGMA method after aligning twelve representative clones with CLUSTALW software (http://www.genome.jp/tools/clustalw). The positions of amino acid changes (V27I, D73G, R387K and L411Q), or deletions (isoleucine 20 (I or Δ), and alanine 376 plus serine 377 (AS or Δ)) are shown. The clone name is composed by CrGluClα, source and Arabic number. Source: Valdivia (Vald), Darwin (Dw) and Errázuriz (Err). Arabic number is the number assigned during the cloning process.

After identifying the sequences of the cDNA ends, we designed specific primers to amplify by PCR the full ORF cDNA. RT-PCRs were carried out using RNA purified from *C. rogercresseyi* from the three geographical sources mentioned above. The amplification yielded products of approximately 1.4 kb (Figure S1) with predicted amino acid sequence showing the general features of the known glutamate gated chloride channels, such as a putative signal peptide preceding the large extracellular N-terminus, two Cys-loops, four transmembrane domains and a shorter C-terminus. In Figure 1, we show a representative clone aligned with the partial sequence available for a putative GluClα from northern hemisphere sea louse *Lepeophterius salmonis*, and the glutamate-gated chloride channel α-subunits of *Drosophila melanogaster* and *Caenorabditis elegans*. The closest to the CrGluCl sequence was that of DmGluClα, which showed a 60% identity and 75% of similarity, with respective figures of 46% and 62% for the comparison with CeGluClα full-length subunit. A more detailed analysis of 33 full-length clones showed some differences in the predicted protein sequence. Predicted sizes ranged from 461 to 464 amino acids and the differences were due to either insertion of one isoleucine at positions 20 (I or Δ) and/or deletion of alanine 376 together with serine 377 (AS or Δ) (see Table S2). In addition, the analysis also revealed 16 amino acid changes (Table S3). To determine if these were genuine differences or whether they originate in PCR errors we amplified smaller, overlapping fragments of the cDNA. Sequencing of the partial clones confirmed the following amino acidic differences, V27I, D73G, R387K and L411Q, plus the above mentioned insertion and deletion. We conclude therefore that they probably represent allelic variants of the protein. Combinations of the precedent variants give rise to 12 different clones whose phylogenetic analysis is shown in Figure 2.

### Functional expression of *C. rogercresseyi* GluClα in *Xenopus laevis* oocytes

To evaluate electrophysiologically the functional properties of *C. rogercresseyi* GluCl we utilised clones CrGluCl-Vald13 and CrGluCl-Vald2 for oocyte expression. CrGluCl-Vald13 was the most abundantly represented of the obtained clones, and CrGluCl-Vald2 contains the greatest number of amino acidic differences compared to CrGluCl-Vald13. As shown in Figure 2 these clones are positioned in the most distant branches of the phylogenetic tree of the allelic variants found. No functional difference whatsoever could be detected between the clones. The results shown below pertain to CrGluCl-Vald13, but to calculate averages these values were pooled with those of CrGluCl-Vald2. The receptor is referred to hereafter simply as CrGluClα.

**Figure 3:**
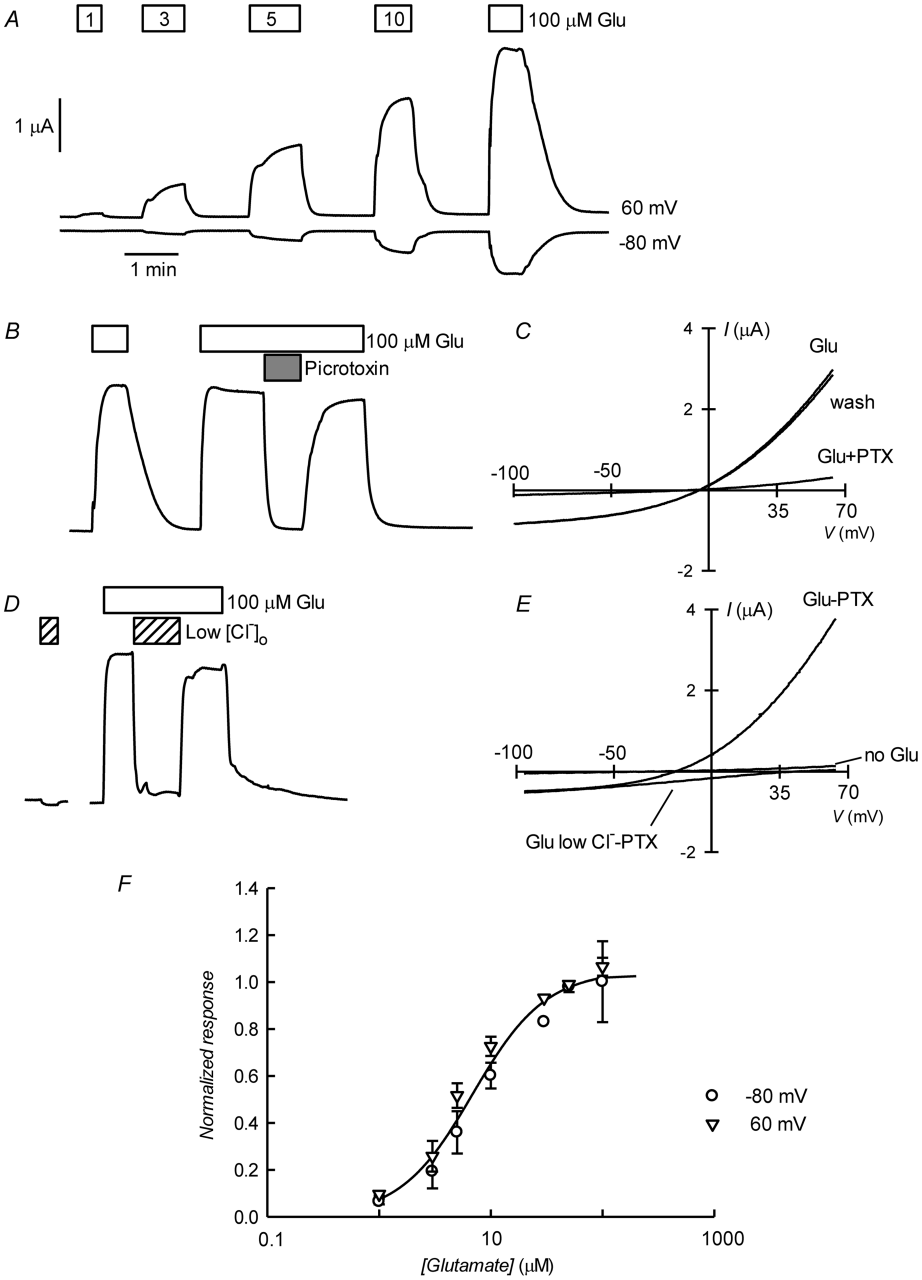
Glutamate-activated picrotoxin-sensitive currents of CrGluClα RNA-injected *Xenopus* oocytes. A. Current traces obtained at the indicated membrane potentials during bath application of glutamate at concentrations from 1 to 100 µM during the times shown in the boxes. Current and time calibration bars shown apply also to recordings shown in panels B and D. B. Picrotoxin (PTX) at 100 µM reversibly abolishes glutamate-evoked current. The trace illustrated was taken at 60 mV. Respective current voltage relations taken from voltage-ramps applied during this experiment are shown in panel C. In panels D and E, it is shown that most of the glutamate evoked outward current is dependent on the presence of extracellular Cl^−^, all but 7.6 mM of which was replaced by gluconate during the time identified as Low [Cl^−^]_o_. The current-voltage relations in E have been corrected by subtraction of the current remaining in 100 µM PTX. Panel F shows a graph summarizing results (means±SEM from 7 experiments) of the concentration-response relationship of the glutamate-sensitive currents. The responses were normalised to the maximal effect evoked by glutamate. The line is a Hill equation fitted simultaneously to both −80 and 60 mV data sets giving values for EC_50_ of 6.89±0.83 µM and n_H_ of 1.33±0.18.

Figure 3A shows current traces obtained using two-electrode voltage-clamp of an oocyte previously injected with CrGluClα RNA. Outward and inward currents were recorded respectively at 60 and −80 mV. Superfusion with increasing concentrations of L-glutamate led to a graded increase in current at both voltages. Figure 3B shows that the glutamate-dependent current could be reversibly inhibited by 100 µM picrotoxin (PTX), a known open ligand-gated chloride channel pore blocker [28], [29]. Average current at 60 mV increased from 0.49±0.043 to 3.09±0.23 µA upon addition of 100 µM glutamate (mean±SEM, n = 18). Addition of PTX decreased the current to 0.48±0.17 µA (n = 14). Current voltage relations taken during addition of 100 µM glutamate before, during and after application of PTX are shown in 3C. The glutamate-dependent current was outwardly rectifying and reversed sign at a depolarized potential compared with the residual current after inhibition with picrotoxin, whose effect was fully reversible. The average reversal potential of the glutamate-induced current was −20±2.2 mV (mean±SEM, n = 6). Figure 3D shows that the current elicited by glutamate was carried by Cl^−^, as extracellular replacement with an impermeant anion sharply reduced outward current (Cl^−^ influx). Current-voltage relations were also taken during glutamate application under normal and low extracellular [Cl^−^]. Figure 3E shows these current-voltage relations that have been corrected for the current remaining in 100 µM PTX, a concentration affording maximal effect of the toxin (Figure S2). The outwardly rectified current was markedly reduced in low external Cl^−^ solution and the reversal potential was shifted in a depolarised direction, as expected for a current carried by Cl^−^. The change in reversal potential ΔE_rev_ was 59±4.2 n = 6 (mean±SEM, n = 6), which did not differ significantly (P = 0.087, one-sample t-test) from the ΔE_rev_ value of 69 mV predicted for perfect selectivity for Cl^−^ over gluconate. Figure 3F shows dose dependence of the glutamate effect measured at −80 and 60 mV. There was no difference between these measurements and a simultaneous fit of a Hill equation to the data gave an EC_50_ of 6.89±0.83 µM and n_H_ of 1.33±0.18.

**Figure 4:**
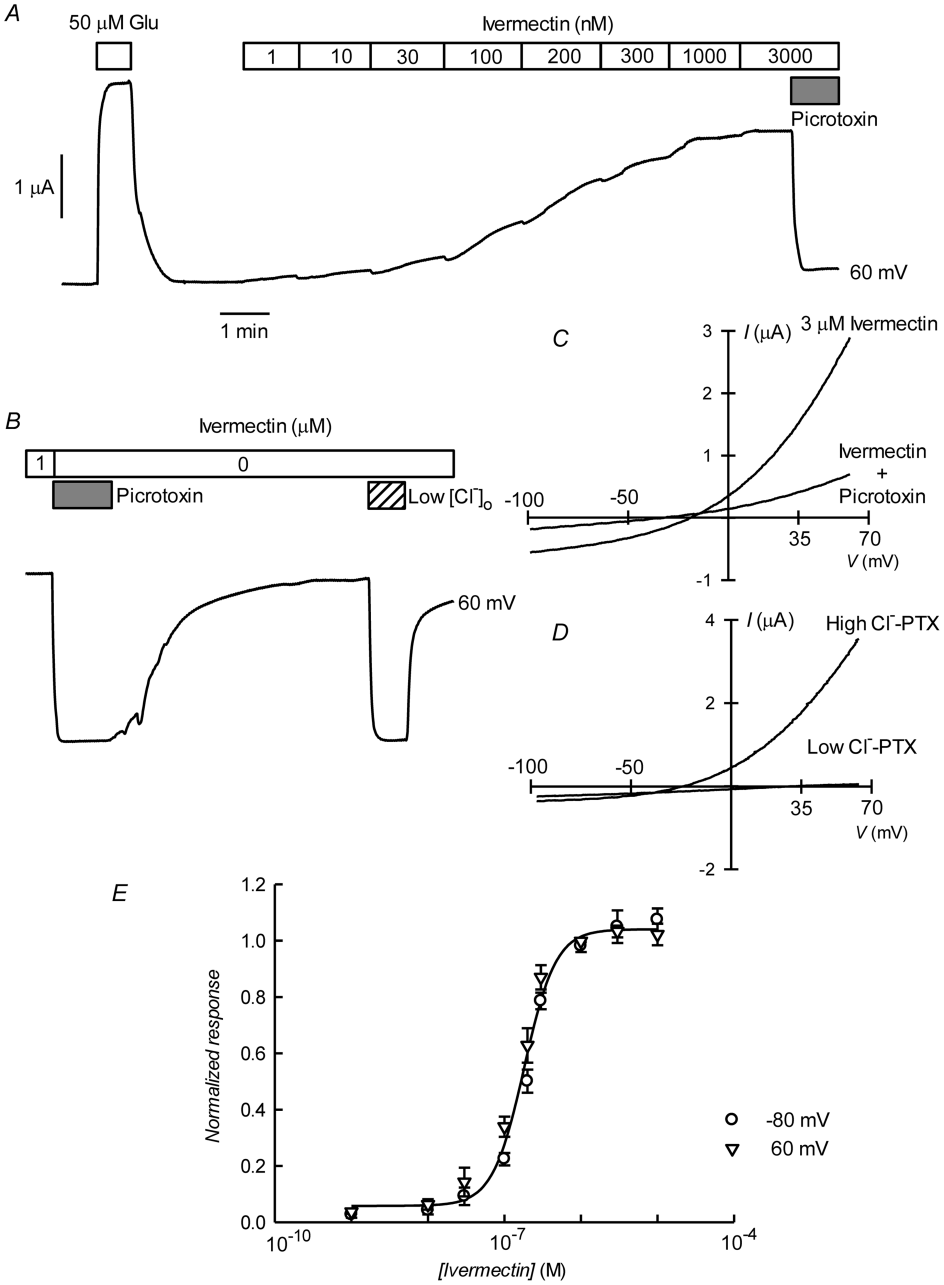
Irreversible activation of CrGluClα by ivermectin. A. Current trace obtained at 60 mV during bath application of 50 µM glutamate and then to ivermectin at concentrations going from 1 to 3000 nM during the times shown in the boxes. The effect of picrotoxin addition at 100 µM is shown at the end of the trace. The trace in panel B shows that whilst ivermectin-induced current blockade by picrotoxin is reversible, the current evoked by the ivermectin is persistent even after prolonged washing. As for the glutamate response, ivermectin-induced outward current was greatly decreased upon decreasing [Cl^−^]_o_ to 7.6 mM. In C and D current voltage relations taken from voltage-ramps applied during experiment in B are shown. Those in D have been corrected by subtracting the current remaining in the presence of 100 µM PTX. E. Dose-response relationship of the Ivermectin-sensitive currents. Data are normalized (mean ± SEM) to the maximal effect of ivermectin and originate in five separate experiments. The line is a Hill equation fitted simultaneously to measurements taken at −80 and 60 mV giving a value for EC_50_ of 181±10 nM, with corresponding n_H_ value of 2.1±0.26.

**Figure 5:**
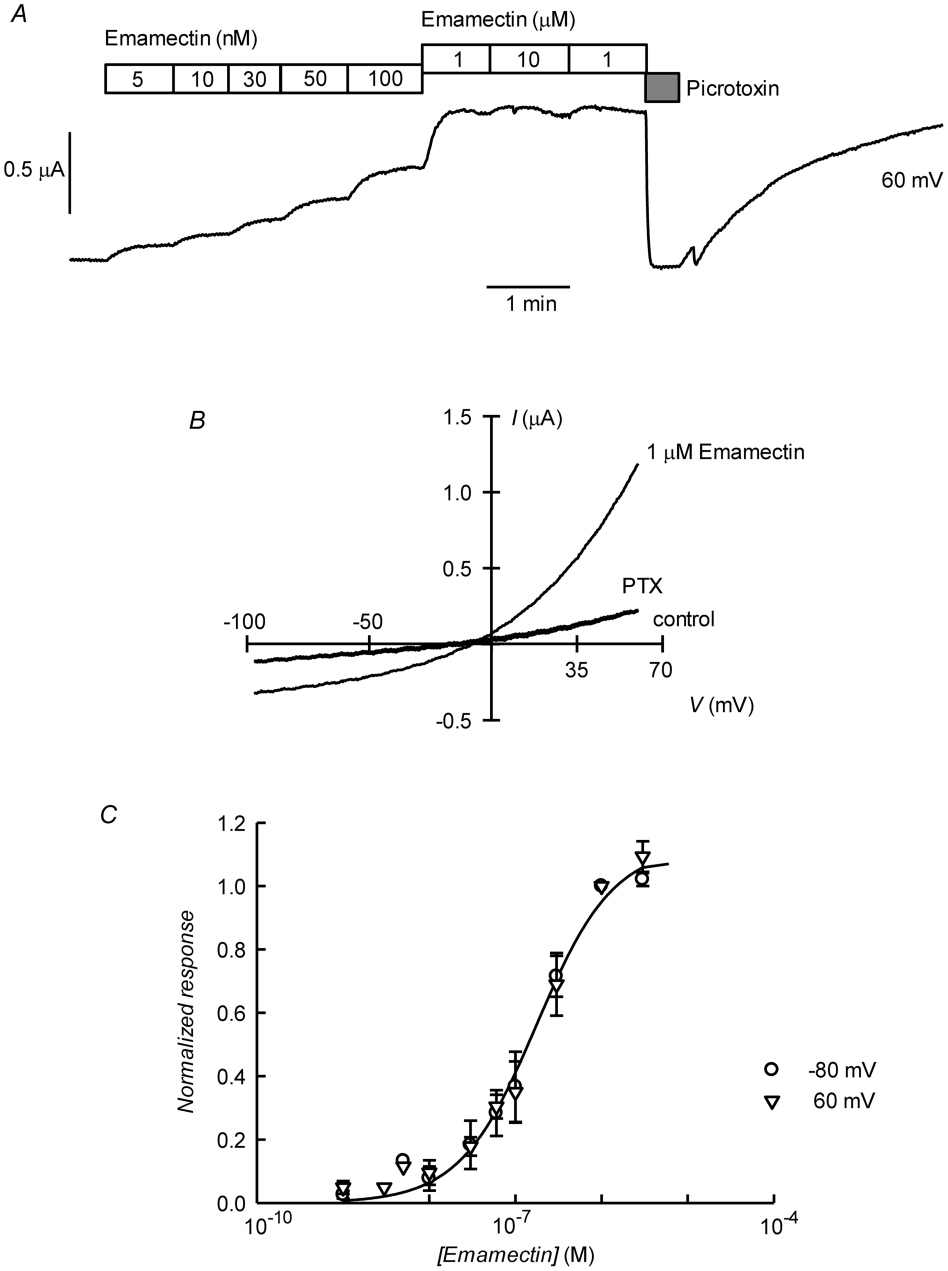
Irreversible activation by emamectin of CrGluClα receptor expressed in *Xenopus* oocytes. A. Current trace obtained at 60 mV during bath application of emamectin at concentrations going from 5 nM to 10 µM during the times shown in the boxes. The effect of picrotoxin addition at 100 µM is also shown immediately after removal of emamectin. Wash out of picrotoxin in the absence of emamectin returns current to the levels attained in the presence of high concentration of emamectin. B: Current-voltage relations taken from voltage-ramps applied during experiment in A. Control denotes current prior to emamectin addition. C. Dose-response relationship of the emamectin-sensitive currents. Data are normalized (mean ± SEM) to the maximal effect of emamectin and originate in seven separate experiments. The line is a Hill equation fitted simultaneously to measurements taken at −80 and 60 mV giving a value for EC_50_ of 202±21 nM and n_H_ 1.1±0.11.

The avermectin family of lactones such as ivermectin are potent and irreversible activators of some ionotropic invertebrate receptors. We have tested the effect of ivermectin on the currents elicited in *Xenopus* oocytes by expression of CrGluClα. Figure 4A shows a recording of CrGluClα current at 60 mV where addition of a supramaximal activating concentration of 50 µM glutamate produced the expected large increase in outward current. Increasing concentrations of ivermectin also elicited a graded increase in outward current readily inhibited by addition of the channel blocker PTX at 100 µM. Figure 4B shows that ivermectin-dependent current was reversibly inhibited by PTX in addition to being dependent on the presence of Cl^−^ in the bath. In six separate experiments ivermectin at 3 µM increased current from 0.39±0.04 to 2.21±0.43 µA, whilst addition of PTX decreased ivermectin-dependent current to 0.40±0.08 µA (means±SEM). The figure also illustrates the irreversibility of ivermectin effect that remains unabated after removal of the drug. Current-voltage relations taken for ivermectin-activated current before and after PTX inhibition are displayed in Figure 4C. The current-voltage relations in Figure 4D have been corrected for the current remaining in 100 µM PTX, in high and low external Cl^−^ solution. The reversal potential shifted in a depolarised direction upon Cl^−^ reduction. The average change in reversal potential ΔE_rev_ was 60±6.7 mV (mean±SEM, n = 4) not significantly different from 69 mV (P = 0.322), consistent with high Cl^−^ selectivity. Emamectin is another member of the avermectin family widely used to combat sea louse infestation in fish aquaculture in its benzoate form. CrGluClα-mediated current was also irreversibly activated by emamectin in a dose-dependent manner (Figure 5A). At 3 µM emamectin, current measured at 60 mV in CrGluClα-expressing oocytes increased from 0.49±0.06 to 1.42±0.13 µA whilst PTX at 100 µM reduced the current to 0.40±0.09 µA (n = 13). Current elicited by a saturating concentration of emamectin was smaller than that elicited by glutamate. Addition of glutamate after activation with emamectin only modestly increased current (Figure S3). Figure 5B shows that the currents stimulated by the avermectins in *Xenopus* oocytes expressing CrGluCl were outwardly rectified. Reversal potential was −21±2.5 (n = 13) mV for emamectin-elicited current, close to the chloride equilibrium potential of *Xenopus* oocytes [30].

Figures 4E and 5C show respectively the dose-response curves for ivermectin and emamectin derived from a number of experiments at 60 and −80 mV. There was no significant difference between values at these two voltages, and when analysed together they gave EC_50_ values for ivermectin and emamectin of 181±10 and 202±21 nM, with corresponding n_H_ values of 2.1±0.26 and 1.1±0.11.

### Molecular modelling of *C. rogercresseyi* GluClα

CrGluClα shares a high percentage of sequence identity with CeGluClα whose X-ray structure has been recently solved [16]. We have used this structure to build a homology model for the *Caligus* receptor to be subsequently used in docking of emamectin and ivermectin followed by molecular dynamics simulations. A view of the resulting modeled structure can be seen in Figure S4. All main features of the reference structure such as secondary structure and side chain conformation were retained in the molecular model of CrGluClα, as expected from the high conservation of sequence particularly in the transmembrane domains.

In order to validate the molecular model and protocol to be used we performed a docking assay with the CeGluClα crystal structure (PDB code 3RHW) and ivermectin. The results produced a position, orientation and interactions of ivermectin with the receptor, with a RMSD of 1.1 Å, very close to those reported in the co-crystallized complex. Interactions included H-bonds with Ser321 and Thr346 in transmembrane domains 2 and 3, and Leu279 of M1 in the adjacent subunit, at respective donor-acceptor distances of 1.9, 1.9 y 2.3 Å. Notice that the numbering used refers to the full-length deduced amino acid sequence (Figure 1) and differs from that used for the crystallized protein that contains some deletions [16]. The CeGluClα-ivermectin docking assay had a GlideScore energy of −8.4 kcal/mol and RMSD of 1.1 Å with respect to the crystal structure.

**Figure 6:**
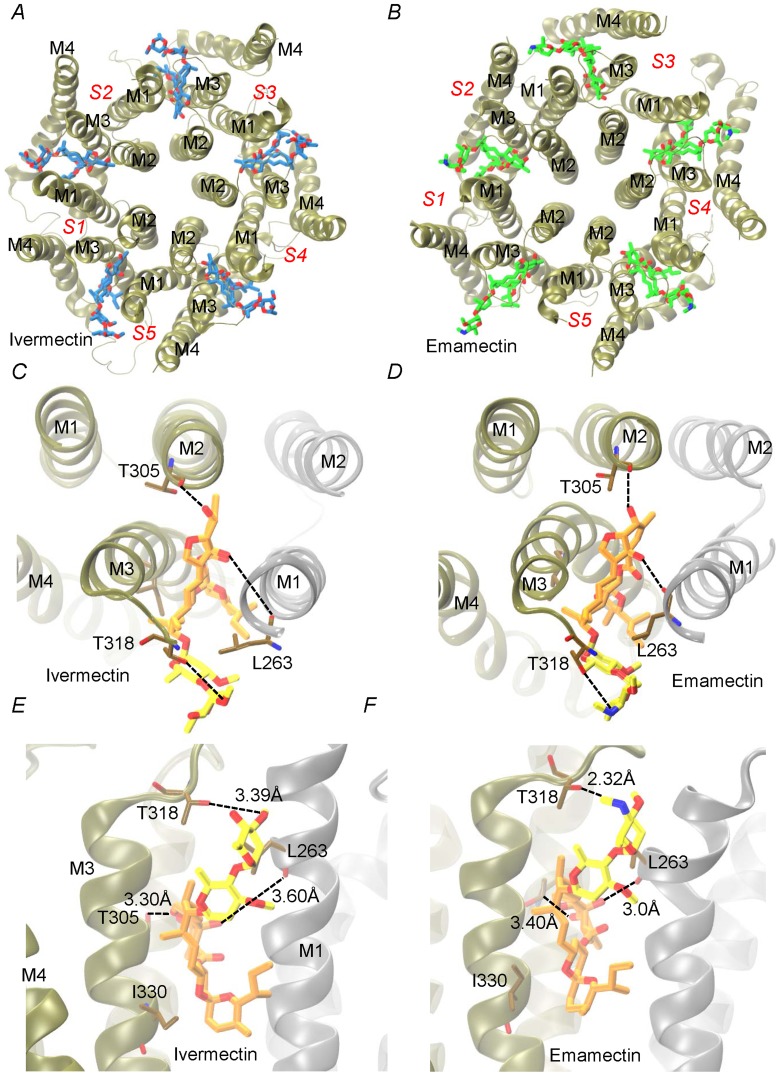
Interaction of emamectin and ivermectin with CrGluClα: results of molecular docking and molecular dynamics experiments. A and B. The final conformation of the transmembrane domains (M1–M4) of CrGluClα for each subunit (S1–S5) after 140 ns MD runs are shown schematically for the receptor after docking with ivermectin (A) or emamectin (B). Emamectin and ivermectin are shown as stick structures. C–F. Higher magnification views of the drug positions in their binding sites. Dotted lines in E and F indicate H-bonds with the residues in stick structures. The view in C and D is from above the channel having removed the extracellular domain. That in E and F is a side-view. Different colours of the transmembrane domains indicate different subunits. Ivermectin and emamectin are displayed with their disaccharide moiety in yellow with the rest of the molecule in orange.

**Figure 7:**
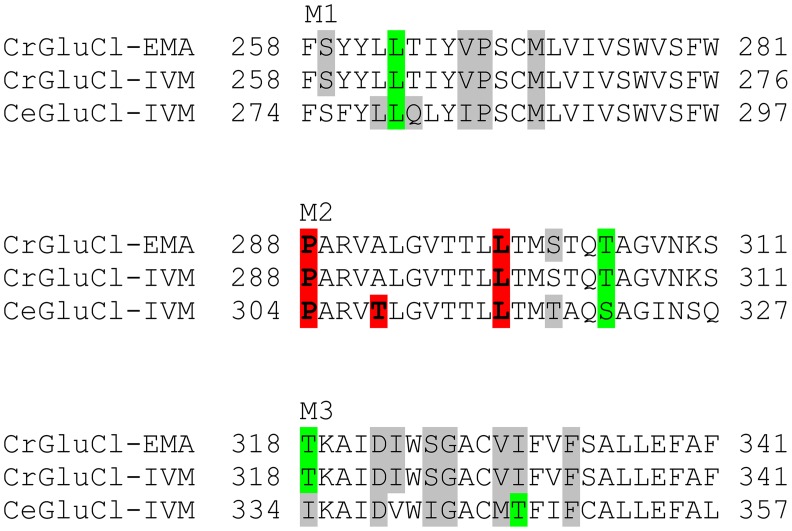
Conservation of residues involved in drug binding in *Caligus* and *C. elegans* GluClα. Residues involved in hydrogen-bonding (green) and van der Waals (grey) interactions of ivermectin or emamectin with CrGluClα (CrGluClα-IVM, CrGluClα-EMA, see text) are compared with those identified in the CeGluClα-ivermectin complex (CeGluClα-IVM) [16]. Only those interactions existing during >70 ns of the 140 ns MD runs are shown. Residues highlighted in red are those apparently involved in creating a hydrophobic seal in the channel pore in the absence of agonists [31].

Docking assays of ivermectin and emamectin employing the CrGluClα model gave the Glide scores values reported in Table 1. Both drugs were seen to be inserted in between subunits and three H-bonds were observed between CrGluClα and ivermectin or emamectin (Table 1). Fig. 6A and B show the positioning of ivermectin and emamectin with respect to the receptor, which occurs between M3 on one subunit and M1 on the neighbouring subunit. Both drugs wedge deeply between M3 and M1, with an -OH group in their cyclohexene ring making H-bond contact with Thr305 of M2 (Figure 6C–F, see Figure S5 for drug structures and identity of groups involved in H-bonding). Two other H-bonds were observed: with the backbone carbonyl oxygen of Leu263 in M1 and with Thr318 at the extracellular end of M3. Residues participating in H-bonding in the CeGluClα-ivermectin crystalographic structure [16] were Leu279 in M1, Ser321 in M2 and Thr346 in M3. Of these, only the first is strictly conserved in CrGluClα (Leu263), with the further two positions occupied by a threonine (305) and an isoleucine (330), as shown in Figure 7. H-bonding at Leu263 and Thr305 of CrGluClα take place with the same positions of ivermectin as corresponding H-bonds at Leu279 and Ser321 in the CeGluClα structure. The Thr318 H-link of CrGluClα is not present in the *C. elegans* structure, where the corresponding position is taken by Ile334. This H-bond of CrGluClα occurs at the extracellular end of M3 and involves a hydroxyl (ivermectin) or an -NH-CH_2_ group (emamectin) in the disaccharide moieties of the drugs (Figure S5). This necessitates a bending upwards in the structures of the drugs to reach the position of Thr318 (Figs. 6C–F).

**Table 1:** Energy and affinity values for the best pose of ivermectin and emamectin in docking assays with CrGluClα molecular model.

Compound	GlideScore (kcal/mol)	H-bonds (n)	H-bonding residues and bond length (Å)		
Ivermectin	−7.9	3	L263 (3.0)	T305 (2.6)	T318 (3.3)
Emamectin	−8.0	3	L263 (3.0)	T305(2.7)	T318 (3.1)

**Figure 8:**
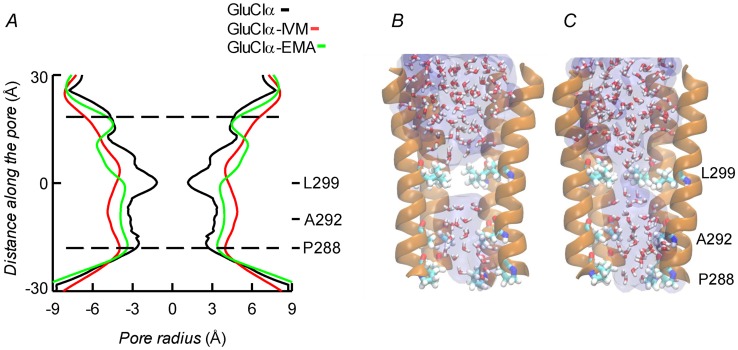
A. Channel pore radius along the z-axis in the receptor alone (black) and in the CrGluClα-emamectin (green) and CrGluClα-ivermectin (red) systems. A length, mainly intramembrane, of the pore is shown. The discontinuous lines show the pore segment lined by M2 α-helices. Values have been taken at the end of MD trajectories. B and C. Lateral views of the pore at time zero (C) and at the end (B) of the MD trajectory for CrGluClα in absence of drugs. Only four M2 helices are shown with the fifth removed for clarity. Residues P288, A292 and L299 are shown in licorice. Water occupancy is shown as licorice and surface.

Molecular dynamics was used to evaluate the stability of the interactions between the protein and the ligands. Three different configurations were run: CrGluClα on its own, the CrGluClα-ivermectin and CrGluClα-emamectin complexes. Figure 6A and B show the positioning of the transmembrane domains and the fit of the drugs after 140 ns MD. Insertion of the drugs into the protein tended to separate helix M3 in one subunit from M1 in an adjacent subunit. It also appears that the top aspect of helix M2 moves away from the pore. These alterations are similar to those described for the crystal structure [16] and in MD studies of CeGluClα and other pentameric ligand-gated ion channels [31], [32]. Minimal diameters measured at the transmembrane portion of the pore at the end of these runs were in the order CrGluClα-ivermectin>CrGluClα-emamectin>CrGluClα (Figure 8A), and are consistent with an open channel configuration for the drug-receptor complexes. Points of narrowest pore diameter in the receptor without drugs occurred at Leu299 and P288 (Figure 8). These correspond to hydrophobic seal amino acids in CeGluClα that are conserved in CrGluClα A third narrowing of the pore (T308 in *C. elegans*) is absent in the *Caligus* receptor where it is replaced by Ala292. Also shown in Figure 8 are structures of the pore of CrGluClα in the absence of drugs at the beginning and the end of the MD simulation. There was a continuous water column at time zero (Figure 8C), that is interrupted by the advance of Leu299 towards the lumen of the pore at the end of the simulation (Figure 8B). The narrowing at P288 did not interrupt the water column.

Identification of the amino acids in the receptor involved in drug interaction considered residues at a distance <3.0 Å during at least 50% of the molecular dynamics time (averaged times of the five interacting drug molecules). According to these criteria 14 residues were involved in ivermectin (15 for emamectin) binding (see Figure 7), including H-bonding Thr305, Thr318, and Leu263, with the rest involved in van der Waals interactions. All five drug molecules remained at their binding sites during the 140 ns MD runs.

### Functional analysis of CrGluCl-T318A mutant

**Figure 9:**
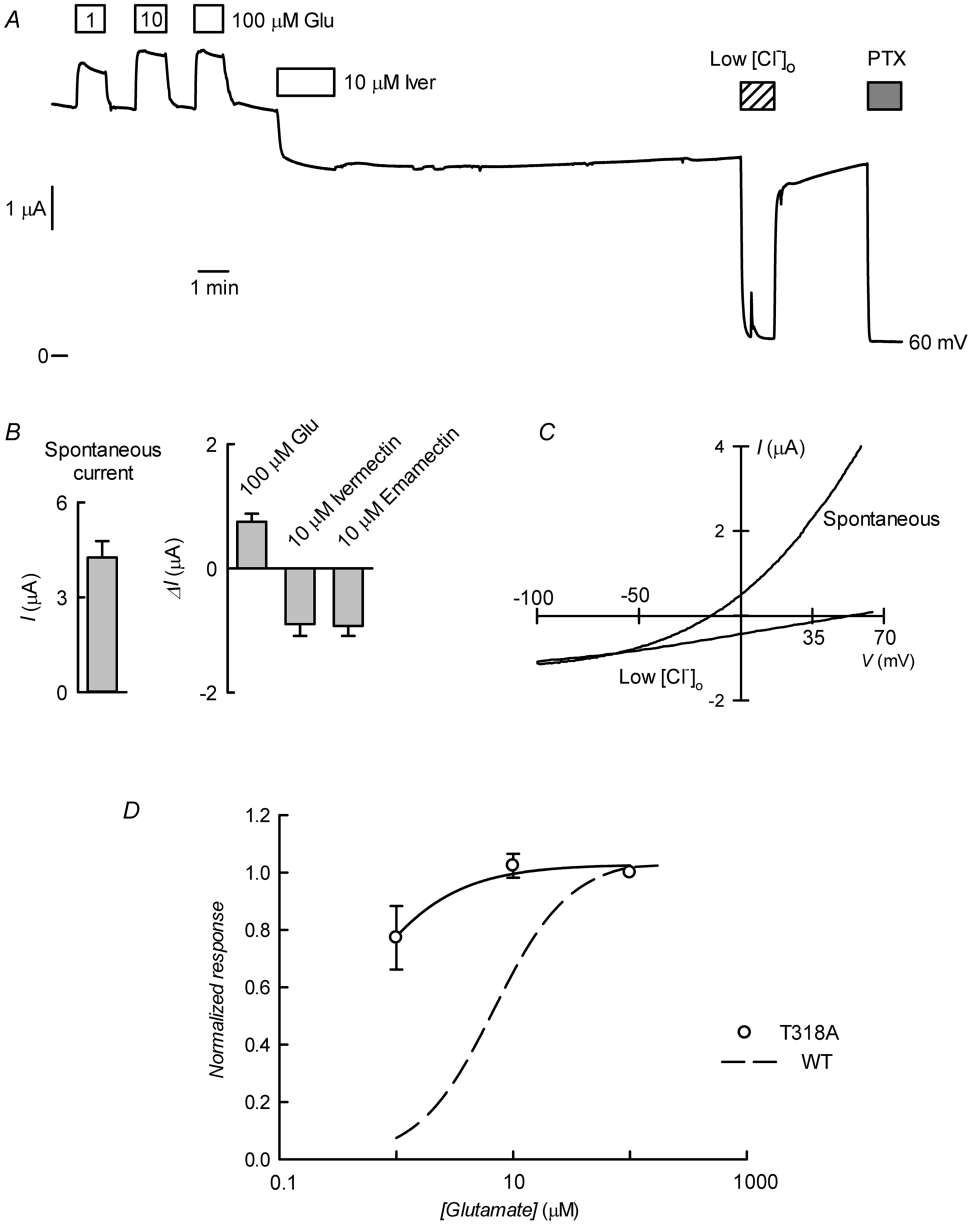
CrGluCl-T318A expression in *Xenopus* oocytes. A. Current trace obtained in a CrGluClα-T318A-expressing oocyte that exhibited high spontaneous current at the indicated membrane potential. Bath application of glutamate at concentrations from 1 to 100 µM took place during the times shown in the boxes. This was followed by exposure to ivermectin, decrease of bath [Cl^−^] to 7.6 mM and addition of 100 µM PTX. B. Average with SEM of spontaneous currents in CrGluClα-T318A-expressing oocytes (n = 7), and increase in current evoked by 100 µM glutamate (n = 7), and the current decrease upon addition of ivermectin (n = 4) or emamectin (n = 5). C. Current voltage relations of spontaneous current exhibited in a CrGluClα-T318A-injected oocyte in normal bath solution and after all but 7.6 mM extracellular Cl^−^ was replaced by gluconate. The current-voltage relations have been corrected by subtraction of the current remaining in 100 µM PTX. D. Concentration-response relationship of the glutamate-sensitive CrGluClα-T318A currents (means±SEM from 7 experiments) normalised to the maximal effect evoked by glutamate. The response of WT CrGluClα is shown for comparison.

The preceding *in silico* analysis of ivermectin and emamectin interaction with CrGluCl revealed several characteristics also found in the crystal structure of CeGluClα bound to ivermectin but also some differences. The most salient divergence emerged in the H-bonds between the avermectins and the receptor. With only two of the three residues involved in CeGluCl conserved in the *Caligus* channel a third, not previously described H-bond acceptor emerged in the form of T318 at the extracellular end of M3. To obtain independent evidence that T318 is involved in the interaction of the avermectins with CrGluCl we mutated this residue to H-bonding-incompetent alanine. Figure 9 shows the result of CrGluClα-T318A expression in *Xenopus* oocytes that exhibited high spontaneous current with addition of glutamate evoking small increases in current and ivermectin inducing partial, irreversible current inhibition (Figure 9A). The current associated to CrGluClα-T318A expression is carried by Cl^−^ ions. This is confirmed by its decrease upon [Cl^−^]_o_ decrease and its blockade by PTX. Figure 9B shows average spontaneous currents in CrGluClα-T318A-expressing oocytes, as well as the increase in evoked by glutamate, and the current decrease observed upon addition of ivermectin or emamectin. Current-voltage relations for spontaneous CrGluClα-T318A activity and that remaining after reduction of extracellular Cl^−^ concentration, after correction for that remaining in the presence of 100 µM PTX, are shown in Figure 9C. The reversal potential, and therefore the resting potential of CrGluClα-T318A-expressing oocytes was −18±1.9 mV (mean±SEM, n = 9). The effect of lowering [Cl^−^]_o_ was to shift the reversal potential by an average ΔE_rev_ of 62±6.9 mV (mean±SEM, n = 3), not significantly different from the 69 mV expected for perfect Cl^−^-selectivity (P = 0.481, one-sample t-test). Average responses to increases in glutamate are shown in Figure 9D and compared to the response of WT CrGluClα. Maximal response was reached at lower glutamate concentrations in the CrGluClα-T318A mutant channel.

**Figure 10:**
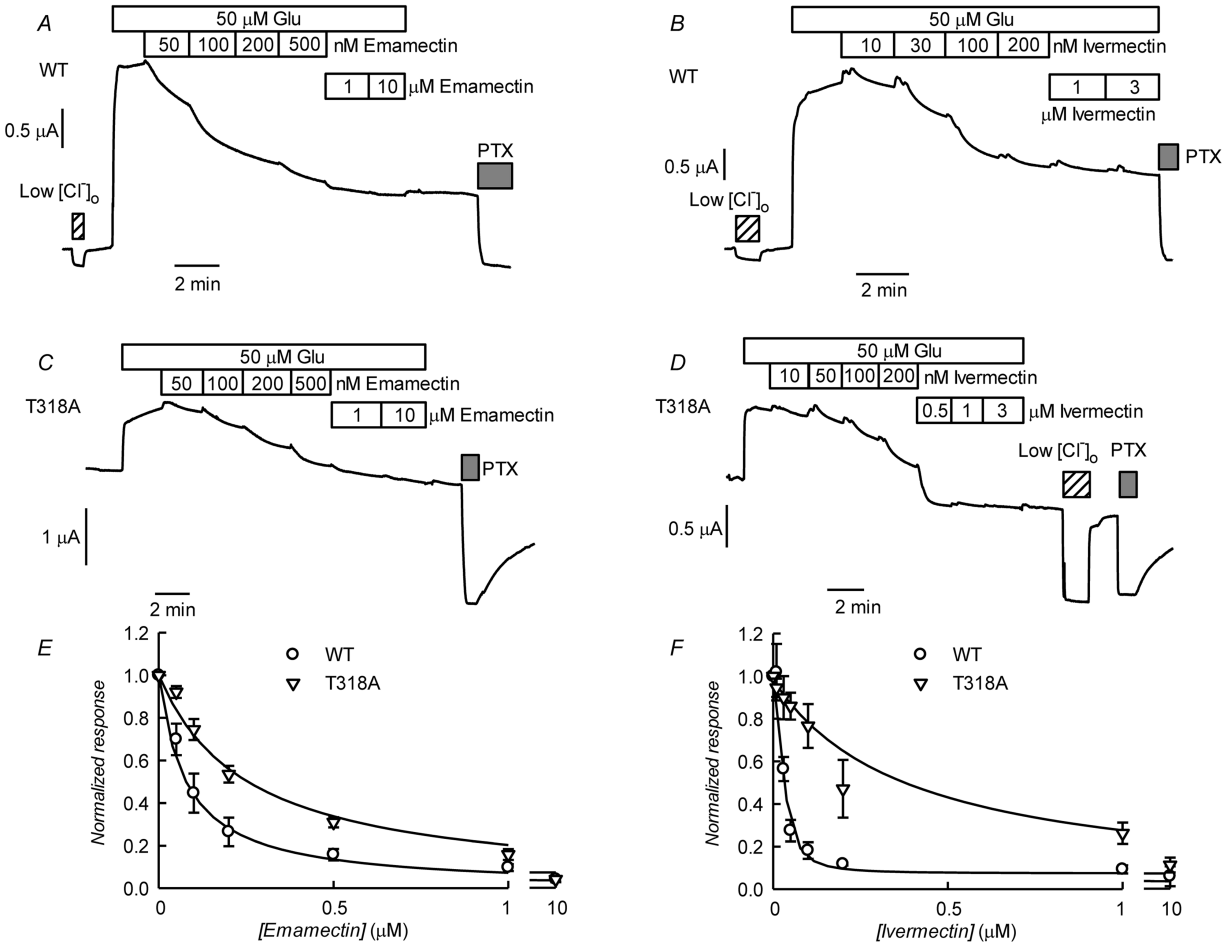
Effect of ivermectin or emamectin upon CrGluClα-T318A-dependent currents. A and B. Effect of increasing concentrations of emamectin and ivermectin on WT CrGluClα-dependent currents. The currents were first activated by addition of 50 µM glutamate and during continuous exposure to glutamate increasing concentrations of emamemctin or ivermectin were added. Reduction of [Cl^−^] to 7.6 mM or addition of 100 mM PTX are also indicated. C and D. Similar experiments performed using the CrGluClα-T318A mutant. E and F. Concentration dependence of the effects of avermectins on WT and T318A mutant CrGluClα receptors. Normalised average responses to emamectin and ivermectin are shown (means±SEM, n = 6 for all experiments shown). Fits of Hill decay functions to the average values are shown by the solid lines.

The data described suggest that CrGluCl-T318A mutant receptor is spontaneously open and is inhibited by emamectin and ivermectin. To study the efficiency of these inhibitory actions we decided to compare the effect of the avermectins in channels having reached what appears to be full opening under the effect of a maximally effective concentration of glutamate. Figure 10A and B show that either emamectin or ivermectin diminished the glutamate-dependent current in receptors activated through addition of 50 µM glutamate in a concentration-dependent manner, reaching a non-zero minimal current at saturating drug (66±9% and 59±8% of the initial current for emamectin and ivermectin respectively, n = 6 for both sets). Similar experiments performed using the CrGluClα-T318A mutant are seen in Figure 10C and D. Maximal effects of emamectin or ivermectin decreased the current activated by glutamate by 66±5 and 60±4% (means±SEM, n = 6 for both experimental sets) respectively. The concentration dependence of the effects of avermectins differed between WT and T318A mutant CrGluClα receptors as shown in the plots of normalised average responses to emamectin and ivermectin in Figure 10E and F. Fits of Hill decay functions to individual experiments gave half maximal inhibitory concentrations for emamectin of 78±22 and 244±31 nM in WT and T318A CrGluClα receptors respectively (means±SEM, n = 6 for both sets of experiments, t-test p = 0.007). Corresponding values for ivermectin inhibition were 30±3.6 and 340±91 nM (means±SEM, n = 6 for both sets of experiments, t-test p = 0.001). Values for n_H_ were not significantly different from unity except for WT ivermectin data that gave an n_H_ value of 1.9±0.4.

## Discussion

Parasitic nematodes, insects and crustaceans are pathogenic or act as disease vectors to man and other vertebrates. The study of the neurobiology of these invertebrates has been spurred by the search of compounds that interfere with their physiological processes and might therefore serve as chemotherapeutic agents for their control. The glutamate-gated chloride channels GluCls are neuronal or muscle receptors that have been described only in invertebrates. This makes them a very interesting pharmacological target for specific agents affecting parasites without interference with the physiology of their vertebrate hosts. Macrocyclic lactones are drugs that activate or modulate the activity of GluCls and have been widely and successfully used as insecticides and antiparasitic agents in human and veterinary medicine and in agriculture [14], [33].

One example of a devastating parasitic problem within an industrial context is the infestation of farmed salmonids with sea lice [3]. MLs ivermectin and, more recently, emamectin have been used successfully to fight these parasites both in the Northern (Norway, Canada, Scotland) and the Southern hemisphere (Chile), where the respective main species involved are *Lepeophtherius salmonis* and *Caligus rogercresseyi*. In both regions, however, ML-resistance has developed by underlying mechanisms that remain essentially unknown [17], [33]–[35]. Possible ways in which drug resistance can arise include a change in the pharmacological target causing the failure of the drug to bind or transduce its binding into the molecular effect, changes in drug metabolism or active removal the drug from the parasite, the host or both perhaps by drug-induced differential gene expression [5], [36].

By analogy with other species of nematodes and insects, it is thought that MLs act on sea louse through irreversible binding to γ-aminobutyric acid and GluCl channels, causing paralysis leading to death. Ivermectin resistance has been associated with mutations in GluCl leading reduced ML-sensitivity in *C. elegans* and *D. Melanogaster* [37]–[39]. This mechanism has not been explored in sea lice as GluCl channels have not been identified molecularly in these species.

An early attempt to obtain a cDNA for GluCl from *L. salmonis* yielded a fragment lacking a start codon [40] which, not surprisingly, failed to produce functional glutamate receptors [41]. The GluCl cDNA isolated here is predicted to result in a 461–464 protein with a putative signal peptide preceding the large extracellular N-terminus, two Cys-loops, four transmembrane domains and a shorter C-terminus. Our sequencing data analysis showed 12 different clones. All the differences were concentrated in the amino end and in the loop located between transmembrane domains 3 and 4, both highly variable regions in glutamate gated chloride channels (see blue arrows in Figure 2). These variable residues have not been reported as involved in glutamate or ivermectin response in other glutamate and ivermectin gated chloride channels so these differences were not predicted to cause changes in the possible functional properties of expressed proteins. Indeed clones 13 and 2 (see Figure 2), the most divergent sequences encountered, gave indistinguishable activities when assayed electrophysiologically after expression in *Xenopus* oocytes.

Closest homology of CrGluClα was with CeGluClα and DmGluClα subunits [11], [42]. Homology with the partial GluCl sequence of Northern hemisphere sea louse *Lepeophtherius salmonis* was also high [40]. Despite sequence homology there are differences in the functional characteristics of CrGluClα and those CeGluClα and DmGluClα. CrGluClα-expressing *Xenopus* oocytes show glutamate-dependent ion currents which, unlike those of DmClGluα [42], do not desensitize. No pretreatment was needed to elicit CrGluClα-mediated currents as is the case for those mediated by CeGluClα that does not show a glutamate response unless previously activated by ivermectin [43]. CrGluClα sensitivity to glutamate is similar to that of DmGluClα [42], with EC_50_ values of 7 and 23 µM respectively, but with the *Caligus* receptor showing little cooperativity (n_H_ 1.3) compared with DmGluClα (n_H_ 2.0). Concerning the effect of ivermectin, both CeGluClα and DmGluClα show activation with respective EC_50_ values of 140 and 40 nM [11], [42] compared with the 180 nM ivermectin EC_50_ of CrGluClα. The effect of ivermectin on CrGluClα was cooperative, with a Hill coefficient of 2.1. This was more reminiscent of that obtained with CeGluClα subunits than with the *Drosophila* α subunit, which shows little cooperativity. Judging by the potency of the ivermectin effect, the receptor of *Caligus* studied here falls within a group of Cys-loop anionic receptors showing the highest affinity ivermectin binding sites [44].

Emamectin benzoate is thought to act in a similar way to ivermectin and has become the chemotherapeutic drug of choice in the treatment of sea louse infestation in the salmon industry [33]. We are not aware, however, of any study of the effect of emamectin on GluCl receptors. Here we show that emamectin activates CrGluClα irreversibly with an EC_50_ of 202 nM and n_H_ of 1.1. The similar potencies for emamectin and ivermectin action together with similarities in structure suggest also a similar mode of action.

Interestingly, the structure of CeGluClα has recently been obtained by X-ray crystallography [16] giving insights into the glutamate and ivermectin binding sites as well as on the site of action of pore inhibitor picrotoxin. The molecular structure of CeGluClα-ivermectin-glutamate reveals nine residues involved in glutamate binding and eight of these are identical in CrGluClα (Figure 1).

The crystal structure of CeGluClα also shows three residues H-bonding with ivermectin, only two of which are conserved in CrGluClα and are seen to H-bond with ivermectin and emamectin in docking assays. A third H-bonding residue in CeGluClα, namely Thr346 that H-links with the spiroketal moiety of ivermectin, is not conserved in CrGluClα. Instead, Thr318 in a region of M3 closer to the extracellular aspect of the channel in CrGluClα H-bonds with the disaccharide end of ivermectin or emamectin. Lack of conservation of CeGluClα Thr346 is also seen in a GluClα receptor from the parasitic nematode *Haemonchus contortus* [45] where the corresponding position is occupied by an alanine. Interestingly this receptor shares with CrGluClα the equivalent of Thr318 and responds to ivermectin in similar fashion as the *Caligus* receptor [46]. The H-bonding between T318 of CrGluClα and the disaccharide moiety of ivermectin and emamectin is novel and requires a bending upwards of the disaccharide moieties of the drug molecules that departs markedly from the topology of ivermectin seen in the crystal structure of CeGluClα. Mutation T318A led to receptors that were spontaneously open, were only slightly enhanced by glutamate and partially inhibited by emamectin or ivermectin. As we noticed in the WT receptor, glutamate and the MLs activate macroscopic currents of different amplitude, with the ML-activated currents significantly smaller than those activated by glutamate. Similarly, lower currents were obtained in the presence of MLs than with glutamate using the CrGluClα-T318A mutant. The potency of drug inhibition of open channels maximally activated by glutamate showed an increased affinity of the avermectins for their binding site in this configuration, perhaps suggesting that splaying of the M1–M3 helices facilitating drug access. Comparison of the EC_50_ values for ivermectin or emamectin inhibition of WT receptors fully activated by glutamate and those activated by mutation T318A shows that drug affinity was decreased by replacement with the H-bonding-incompetent Ala.

We have not yet a testable hypothesis of a possible mechanism for the T318A mutation-induced activation. We speculate that breaking a putative intramolecular H-bond in which T318 plays an acceptor role, either by the mutation or by action of the ML drugs, could lead to opening of CrGluClα receptor. Interestingly a disease-causing mutation at an equivalent site (V280M) in a glycine receptor leads to spontaneously active channels [47]. It is postulated that V280 might interact with residues in M1 to stabilize the closed state of the channel. Our results, although not entirely understood, point to the importance of T318 in the interaction of emamectin and ivermectin with the *Caligus* receptor. Perhaps future molecular simulations of the T318A receptor could clarify the role of this residue that appears to play an important part in the channel open-closed equilibrium.

Avermectin resistance through mutation in glutamate receptors has been described. A naturally occurring mutation (G323D) at M3 in a *Tetranychus urticae* GluCl (corresponding to Gly326 in CrGluClα) markedly increases abamectin LD_50_ [48]. Human GlyRα1 receptor has an alanine at the equivalent position (288) and shows low sensitivity to ivermectin, which becomes 50-fold higher in A288G-HsGlyRα1. The reciprocal mutation in HcGluα3B, G329A, converts this normally high ivermectin-sensitive receptor into a relatively resistant one [39]. This glycine residue, conserved in GluClα channels, lies at the M3 domain facing the drug binding cavity and it is conceivable that its mutation alters M3 flexibility and the interaction of avermectins with the receptor. This residue dubbed, M3-Gly, appears essential for high ivermectin affinity and is highly conserved in GluCl receptors [44].Mutation P299S, corresponding to P313 in CrGluClα, also markedly decreases the ability of ivermectin to activate DmGluClα [38]. The proline residue is located at the M2–M3 loop that is thought to be stabilised by ivermectin in a conformation favouring the open state [16]. Its mutation might interfere with channel opening by avermectins without a direct interaction with the drugs.

Van der Waals interactions were identified with twelve residues of CeGluClα, with nine of those identical or similar in the *Caligus* receptor (see Figure 7). Although not studied here in detail it appears that the mechanism whereby ivermectin, and emamectin, maintains CeGluClα open is by intercalating between subunits leading to the separation of helices M1 and M3 of neighbouring subunits [16]. As pointed out by Hibbs and Gouaux [16], the site defined by the binding of avermectins in GluClα receptors appears equivalent to that targeted in other pentameric ligand-gated ion channels by modulators such as alcohol and anesthetics [49]. Another structural characteristic of receptors defined has having high ivermectin sensitivity has been proposed to be the possibility of making numerous H-bonding interactions in the vicinity of S321 that was identified as H-binding in the crystal structure of the *C. elegans* channel [16]. These possible H-bond partners are M2 T318, S321 and N325, and Q320 and Q280 of M2, and M1 in the neighbouring subunit of the *C. elegans* receptor [44]. Their corresponding residues in the *Caligus* channel are S302, T305 (identified as H-bond partner in our calculations), N309/Q304/T264, thus conserving the capability for H-binding in this receptor and possibly contributing to explain its affinity for ivermectin and emamectin.

Our docking and MD calculations suggest that despite using the same general binding pocket, ivermectin and emamectin are seen at the *Caligus* receptor in a different conformation than that revealed by the crystal of the *C. elegans* receptor-ivermectin complex and form a different three-dimensional network of H-bonds [16]. This observation, in addition to requiring more work for its confirmation and better understanding, identifies a different type of drug binding site in CrGluClα that could admit new types of activity-modifying molecules.

We have presented here the first report of a full-length ionotropic glutamate-gated GluClα from the salmon parasite *Caligus rogercresseyi*. Our functional expression data strongly suggest that CrGluClα is an important target for avermectins used in the treatment parasitic infestation in salmon and trout and opens the way for ascertaining a possible mechanism of increasing resistance problems dogging aquaculture industry worldwide. Molecular modeling and docking assays as we demonstrate here could help in the design of new, more efficient drugs with functional expression of the receptor allowing a first stage of testing of their efficacy.

## Supporting Information

Figure S1 Lanes are: 1: RT (+), 2: RT (−), 3: 1 kb ladder, and 4: water control.
(PDF)

Figure S2 Effect of picrotoxin (PTX) on CrGluClα-mediated currents. A. Current measured at 60 mV in an oocyte expressing the *Caligus* receptor. Additions to the bathing medium are shown in the boxes above. B. Concentration dependence of PTX. The points correspond to data from four separate experiments. The EC_50_ of 3.2 µM is the mean of hyperbolic fits to the individual data.
(PDF)

Figure S3 Comparison of the effects of saturation concentrations of glutamate and emamectin on CrGluClα-mediated current measured after expression in Xenopus oocytes. Data are means ± SEM of number of experiments given in brackets.
(PDF)

Figure S4 Homology model of *Caligus rogercresseyi* glutamate receptor CrGluClα. A: Vista lateral view. B. Upper view from the extracellular side. The protein is shown in Newcartoon drawing method. Transmembrane α-helices (M1–M4) are in blue, extracelluar β-sheets are shown in red, and coils in yellow.
(PDF)

Figure S5 Chemical structures of ivermectin and emamectin. Sites involved in H-bond interactions with CrGluα as suggested by the docking assays are indicated. M2 and M3 belong to one subunit whilst M1 is in the neighbouring subunit. Images are from the PubChem (http://pubchem.ncbi.nlm.nih.gov/) database.
(PDF)

Table S1 Primers used in PCR reactions at various stages of the cloning of *C. rogercresseyi* GluClα receptor.
(PDF)

Table S2 Summary of amino acid differences in full length clones. The upper row shows the two possible options of amino acid or deletions with their respective positions. The bottom row shows the number of times they were found. Highlighted in grey are shown the groups that were selected as different branches to build the phylogenetic tree of Figure 2 in the main text.
(PDF)

Table S3 Summary of amino acid differences in partial clones. The differences, positions and number of times they occurred in the study are shown as for Table S2. 31, 29 and 34 clones were analyzed respectively for 5′, middle and 3′ fragments.
(PDF)

## References

[1] Costello MJ (2006) Ecology of sea lice parasitic on farmed and wild fish. Trends Parasitol 22: 475–483 S1471-4922(06)00211-X [pii]; 10.1016/j.pt.2006.08.006 [doi] 16920027

[2] Boxshall GA, Bravo S (2000) On the identity of the common *Caligus* (Copepoda: Siphonostomatoida: Caligidae) from salmonid netpen systems in southern Chile. Contrib Zool 69: 137–146.

[3] Torrissen O, Jones S, Asche F, Guttormsen A, Skilbrei OT, et al (2013) Salmon lice-impact on wild salmonids and salmon aquaculture. J Fish Dis 36: 171–194 10.1111/jfd.12061 [doi] 23311858 PMC3675643

[4] Dent JA, Davis MW, Avery L (1997) avr-15 encodes a chloride channel subunit that mediates inhibitory glutamatergic neurotransmission and ivermectin sensitivity in *Caenorhabditis elegans* . EMBO J 16: 5867–5879 10.1093/emboj/16.19.5867 [doi] 9312045 PMC1170218

[5] Wolstenholme AJ, Fairweather I, Prichard R, von Samson-Himmelstjerna G, Sangster NC (2004) Drug resistance in veterinary helminths. Trends Parasitol 20: 469–476 10.1016/j.pt.2004.07.010 [doi];S1471-4922(04)00201-6 [pii] 15363440

[6] Jones AK, Sattelle DB (2006) The cys-loop ligand-gated ion channel superfamily of the honeybee, *Apis mellifera* . Invert Neurosci 6: 123–132 10.1007/s10158-006-0026-y [doi] 16902773

[7] Jones AK, Sattelle DB (2007) The cys-loop ligand-gated ion channel gene superfamily of the red flour beetle, *Tribolium castaneum* . BMC Genomics 8: 327 1471-2164-8-327 [pii]; 10.1186/1471-2164-8-327 [doi] 17880682 PMC2064938

[8] Jones AK, Sattelle DB (2008) The cys-loop ligand-gated ion channel gene superfamily of the nematode, *Caenorhabditis elegans* . Invert Neurosci 8: 41–47 10.1007/s10158-008-0068-4 [doi] 18288508 PMC2257991

[9] Knipple DC, Soderlund DM (2010) The ligand-gated chloride channel gene family of *Drosophila melanogaster* . Pest Biochem Physiol 97: 140–148.

[10] Raymond V, Sattelle DB (2002) Novel animal-health drug targets from ligand-gated chloride channels. Nat Rev Drug Discov 1: 427–436 10.1038/nrd821 [doi] 12119744

[11] Cully DF, Vassilatis DK, Liu KK, Paress PS, Van der Ploeg LH, et al (1994) Cloning of an avermectin-sensitive glutamate-gated chloride channel from *Caenorhabditis elegans* . Nature 371: 707–711 10.1038/371707a0 [doi] 7935817

[12] Vassilatis DK, Arena JP, Plasterk RH, Wilkinson HA, Schaeffer JM, et al (1997) Genetic and biochemical evidence for a novel avermectin-sensitive chloride channel in *Caenorhabditis elegans*. Isolation and characterization. J Biol Chem 272: 33167–33174. 9407104 10.1074/jbc.272.52.33167

[13] Cheeseman CL, Delany NS, Woods DJ, Wolstenholme AJ (2001) High-affinity ivermectin binding to recombinant subunits of the Haemonchus contortus glutamate-gated chloride channel. Mol Biochem Parasitol 114: 161–168 S0166685101002584 [pii] 11378196 10.1016/s0166-6851(01)00258-4

[14] Wolstenholme AJ (2012) Glutamate-gated chloride channels. J Biol Chem 287: 40232–40238 R112.406280 [pii]; 10.1074/jbc.R112.406280 [doi] 23038250 PMC3504739

[15] Cully DF, Wilkinson H, Vassilatis DK, Etter A, Arena JP (1996) Molecular biology and electrophysiology of glutamate-gated chloride channels of invertebrates. Parasitology 113 Suppl: S191–S200. 9051935 10.1017/s0031182000077970

[16] Hibbs RE, Gouaux E (2011) Principles of activation and permeation in an anion-selective Cys-loop receptor. Nature 474: 54–60 nature10139 [pii]; 10.1038/nature10139 [doi] 21572436 PMC3160419

[17] Bravo S, Sevatdal S, Horsberg TE (2008) Sensitivity assessment of *Caligus rogercresseyi* to emamectin benzoate in Chile. Aquaculture 282: 7–12.

[18] Huang JC, Chen F (2006) Simultaneous amplification of 5′ and 3′ cDNA ends based on template-switching effect and inverse PCR. Biotechniques 40: 187–189 000112051 [pii] 16526408 10.2144/000112051

[19] Sali A, Blundell TL (1993) Comparative protein modelling by satisfaction of spatial restraints. J Mol Biol 234: 779–815. 8254673 10.1006/jmbi.1993.1626

[20] Phillips JC, Braun R, Wang W, Gumbart J, Tajkhorshid E, et al (2005) Scalable molecular dynamics with NAMD. J Comput Chem 26: 1781–1802 10.1002/jcc.20289 [doi] 16222654 PMC2486339

[21] Glide (2012) 5.8 [computer program]. New York NY USA: Schrödinger LLC.

[22] Maestro (2012) 9.3 [computer program]. New York NY USA: Schrödinger LLC.

[23] LigPrep (2012) 2.5 [computer program]. New York NY USA: Schrödinger LLC.

[24] Sastry GM, Adzhigirey M, Day T, Annabhimoju R, Sherman W (2013) Protein and ligand preparation: parameters, protocols, and influence on virtual screening enrichments. J Comput Aided Mol Des 27: 221–234 10.1007/s10822-013-9644-8 [doi] 23579614

[25] Jorgensen WL, Chandrasekhar J, Madura JD, Impey RW, Klein MI (1983) Comparison of simple potential functions for simulating liquid water. J Chem Phys 79: 926–935.

[26] Tuckerman ME, Martyna GJ, Berne BJ (1992) Reversible multiple time scale molecular dynamics. J Chem Phys 97: 1990–2001.

[27] Smart OS, Neduvelil JG, Wang X, Wallace BA, Sansom MS (1996) HOLE: a program for the analysis of the pore dimensions of ion channel structural models. J Mol Graph 14: 354–60, 376 S026378559700009X [pii] 9195488 10.1016/s0263-7855(97)00009-x

[28] Takeuchi A, Takeuchi N (1969) A study of the action of picrotoxin on the inhibitory neuromuscular junction of the crayfish. J Physiol 205: 377–391. 5357245 10.1113/jphysiol.1969.sp008972 PMC1348609

[29] Etter A, Cully DF, Liu KK, Reiss B, Vassilatis DK, et al (1999) Picrotoxin blockade of invertebrate glutamate-gated chloride channels: subunit dependence and evidence for binding within the pore. J Neurochem 72: 318–326. 9886084

[30] Miledi R, Parker I (1984) Chloride current induced by injection of calcium into Xenopus oocytes. J Physiol 357: 173–183. 6096530 10.1113/jphysiol.1984.sp015495 PMC1193253

[31] Cheng MH, Coalson RD (2012) Energetics and ion permeation characteristics in a glutamate-gated chloride (GluCl) receptor channel. J Phys Chem B 116: 13637–13643 10.1021/jp3074915 [doi] 23088363

[32] Calimet N, Simoes M, Changeux JP, Karplus M, Taly A, et al (2013) A gating mechanism of pentameric ligand-gated ion channels. Proc Natl Acad Sci U S A 110: E3987–E3996 1313785110 [pii]; 10.1073/pnas.1313785110 [doi] 24043807 PMC3801054

[33] Horsberg TE (2012) Avermectin use in aquaculture. Curr Pharm Biotechnol 13: 1095–1102 BSP/CPB/E-Pub/0000125 [pii] 22039799 10.2174/138920112800399158

[34] Bravo S, Nunez M, Silva MT (2013) Efficacy of the treatments used for the control of Caligus rogercresseyi infecting Atlantic salmon, Salmo salar L., in a new fish-farming location in Region XI, Chile. J Fish Dis 36: 221–228 10.1111/jfd.12023 [doi] 23347203

[35] Lees F, Baillie M, Gettinby G, Revie CW (2008) The efficacy of emamectin benzoate against infestations of Lepeophtheirus salmonis on farmed Atlantic salmon (Salmo salar L) in Scotland, 2002–2006. PLoS ONE 3: e1549 10.1371/journal.pone.0001549 [doi] 18253496 PMC2212131

[36] Igboeli OO, Burka JF, Fast MD (2013) Sea lice population and sex differences in P-glycoprotein expression and emamectin benzoate resistance on salmon farms in the Bay of Fundy, New Brunswick, Canada. Pest Manag Sci 10.1002/ps.3620 [doi] 23913539

[37] Dent JA, Smith MM, Vassilatis DK, Avery L (2000) The genetics of ivermectin resistance in *Caenorhabditis elegans* . Proc Natl Acad Sci U S A 97: 2674–2679 97/6/2674 [pii] 10716995 10.1073/pnas.97.6.2674 PMC15988

[38] Kane NS, Hirschberg B, Qian S, Hunt D, Thomas B, et al (2000) Drug-resistant Drosophila indicate glutamate-gated chloride channels are targets for the antiparasitics nodulisporic acid and ivermectin. Proc Natl Acad Sci U S A 97: 13949–13954. 11095718 10.1073/pnas.240464697 PMC17681

[39] Lynagh T, Lynch JW (2010) A glycine residue essential for high ivermectin sensitivity in Cys-loop ion channel receptors. Int J Parasitol 40: 1477–1481 S0020-7519(10)00281-X [pii]; 10.1016/j.ijpara.2010.07.010 [doi] 20713056

[40] Tribble ND, Burka JF, Kibenge FS (2007) Identification of the genes encoding for putative gamma aminobutyric acid (GABA) and glutamate-gated chloride channel (GluCl) alpha receptor subunits in sea lice (*Lepeophtheirus salmonis*). J Vet Pharmacol Ther 30: 163–167 JVP823 [pii]; 10.1111/j.1365-2885.2007.00823.x [doi] 17348903

[41] Young PD (2008) Binding characteristics of emamectin benzoate to the putative glutamate-gated chloride channels of *Lepeophtheirus salmonis* [dissertation]. University of Prince Edward Island, Canada. 108 p.

[42] Cully DF, Paress PS, Liu KK, Schaeffer JM, Arena JP (1996) Identification of a *Drosophila melanogaster* glutamate-gated chloride channel sensitive to the antiparasitic agent avermectin. J Biol Chem 271: 20187–20191. 8702744 10.1074/jbc.271.33.20187

[43] Etter A, Cully DF, Schaeffer JM, Liu KK, Arena JP (1996) An amino acid substitution in the pore region of a glutamate-gated chloride channel enables the coupling of ligand binding to channel gating. J Biol Chem 271: 16035–16039. 8663156 10.1074/jbc.271.27.16035

[44] Lynagh T, Lynch JW (2012) Ivermectin binding sites in human and invertebrate Cys-loop receptors. Trends Pharmacol Sci 33: 432–441 S0165-6147(12)00074-0 [pii]; 10.1016/j.tips.2012.05.002 [doi] 22677714

[45] Forrester SG, Hamdan FF, Prichard RK, Beech RN (1999) Cloning, sequencing, and developmental expression levels of a novel glutamate-gated chloride channel homologue in the parasitic nematode Haemonchus contortus. Biochem Biophys Res Commun 254: 529–534 S0006-291X(98)90106-1 [pii]; 10.1006/bbrc.1998.0106 [doi] 9920773

[46] Forrester SG, Prichard RK, Dent JA, Beech RN (2003) Haemonchus contortus: HcGluCla expressed in Xenopus oocytes forms a glutamate-gated ion channel that is activated by ibotenate and the antiparasitic drug ivermectin. Mol Biochem Parasitol 129: 115–121 S0166685103001026 [pii] 12798512 10.1016/s0166-6851(03)00102-6

[47] Bode A, Lynch JW (2013) Analysis of hyperekplexia mutations identifies transmembrane domain rearrangements that mediate glycine receptor activation. J Biol Chem 288: 33760–33771 M113.513804 [pii]; 10.1074/jbc.M113.513804 [doi] 24097980 PMC3837120

[48] Kwon DH, Yoon KS, Clark JM, Lee SH (2010) A point mutation in a glutamate-gated chloride channel confers abamectin resistance in the two-spotted spider mite, Tetranychus urticae Koch. Insect Mol Biol 19: 583–591 IMB1017 [pii]; 10.1111/j.1365-2583.2010.01017.x [doi] 20522121

[49] Corringer PJ, Poitevin F, Prevost MS, Sauguet L, Delarue M, et al (2012) Structure and pharmacology of pentameric receptor channels: from bacteria to brain. Structure 20: 941–956 S0969-2126(12)00184-0 [pii]; 10.1016/j.str.2012.05.003 [doi] 22681900

