# Correction: Identification and Functional Expression of a Glutamate- and Avermectin-Gated Chloride Channel from *Caligus rogercresseyi*, a Southern Hemisphere Sea Louse Affecting Farmed Fish

**DOI:** 10.1371/journal.ppat.1004494

**Published:** 2014-10-13

**Authors:** The PLOS Pathogens Staff

The fifth author's name is spelled incorrectly. The correct name is: Fernando D. González-Nilo.

The secondary affiliation for the 5^th^ author is not indicated. Fernando D. González-Nilo is also affiliated with Centro Interdisciplinario de Neurociencias de Valparaiso, CINV, Valparaiso, Chile.
