# Supplementary figures and images for: Identification and Functional Expression of a Glutamate- and Avermectin-Gated Chloride Channel from Caligus rogercresseyi, a Southern Hemisphere Sea Louse Affecting Farmed Fish
Source: PLoS Pathog. 2014 Sep 25;10(9):e1004402. doi: 10.1371/journal.ppat.1004402 (PMC4177951; doi:10.1371/journal.ppat.1004402)

Figure S1

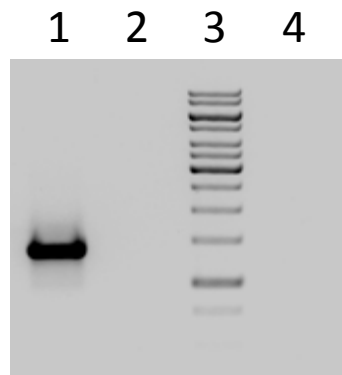

Figure S1. Lanes are: 1: RT (+), 2: RT (-), 3: 1kb ladder, and 4: water control.

Supplement: Figure S1 — Lanes are: 1: RT (+), 2: RT (−), 3: 1 kb ladder, and 4: water control. (PDF) [file ppat.1004402.s001.pdf]
