# Supplementary material for: Identification and Functional Expression of a Glutamate- and Avermectin-Gated Chloride Channel from Caligus rogercresseyi, a Southern Hemisphere Sea Louse Affecting Farmed Fish
Source: PLoS Pathog. 2014 Sep 25;10(9):e1004402. doi: 10.1371/journal.ppat.1004402 (PMC4177951; doi:10.1371/journal.ppat.1004402)
Supplement: Figure S2 — Effect of picrotoxin (PTX) on CrGluClα-mediated currents. A. Current measured at 60 mV in an oocyte expressing the Caligus receptor. Additions to the bathing medium are shown in the boxes above. B. Concentration dependence of PTX. The points correspond to data from four separate experiments. The EC50 of 3.2 µM is the mean of hyperbolic fits to the individual data. (PDF) [file ppat.1004402.s002.pdf]

Figure S2

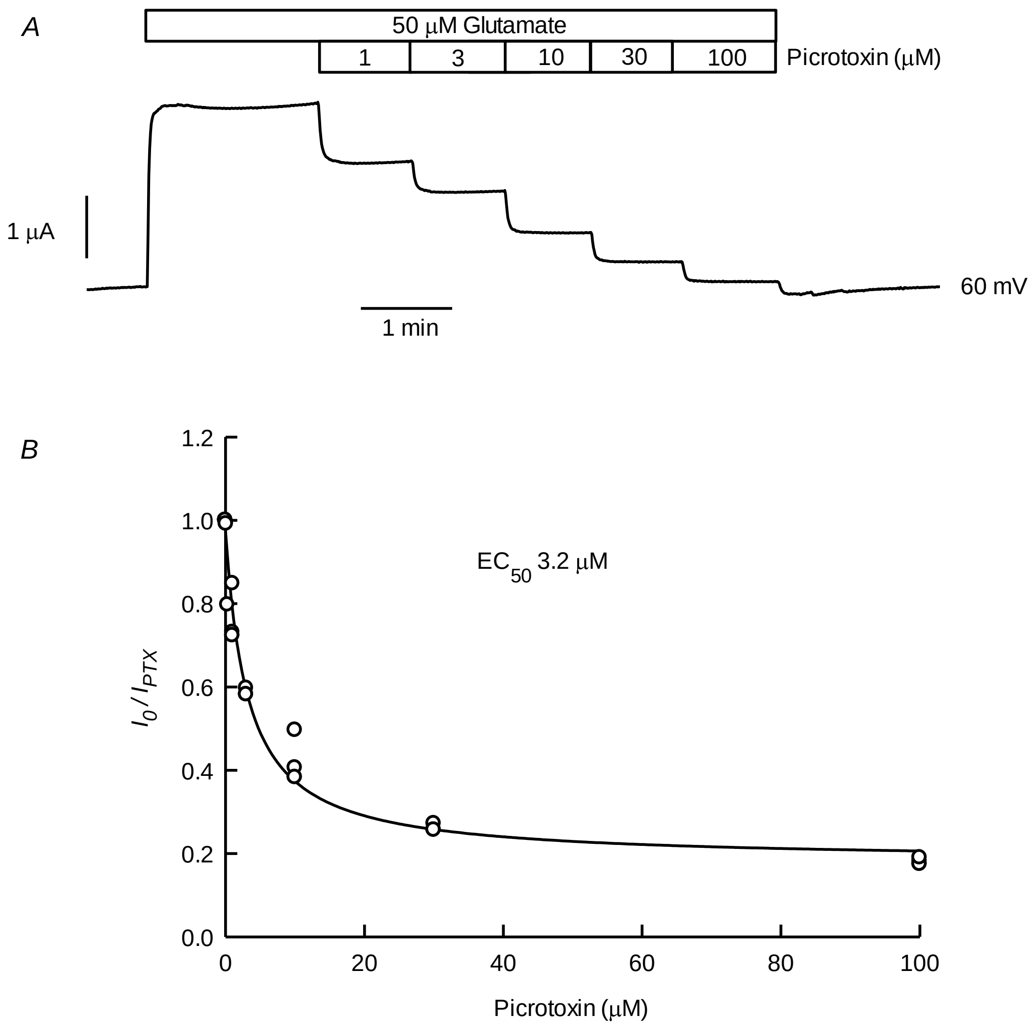

Figure S2. Effect of picrotoxin (PTX) on CrGluClα-mediated currents. A. Current measured at 60 mV in an oocyte expressing the *Caligus* receptor. Additions to the bathing medium are shown in the boxes above. B. Concentration dependence of PTX effect. The points correspond to data from four separate experiments. The EC_{50} of 3.2 μM is the mean of hyperbolic fits to the individual data.
