# Supplementary material for: Identification and Functional Expression of a Glutamate- and Avermectin-Gated Chloride Channel from Caligus rogercresseyi, a Southern Hemisphere Sea Louse Affecting Farmed Fish
Source: PLoS Pathog. 2014 Sep 25;10(9):e1004402. doi: 10.1371/journal.ppat.1004402 (PMC4177951; doi:10.1371/journal.ppat.1004402)
Supplement: Figure S3 — Comparison of the effects of saturation concentrations of glutamate and emamectin on CrGluClα-mediated current measured after expression in Xenopus oocytes. Data are means ± SEM of number of experiments given in brackets. (PDF) [file ppat.1004402.s003.pdf]

Figure S3

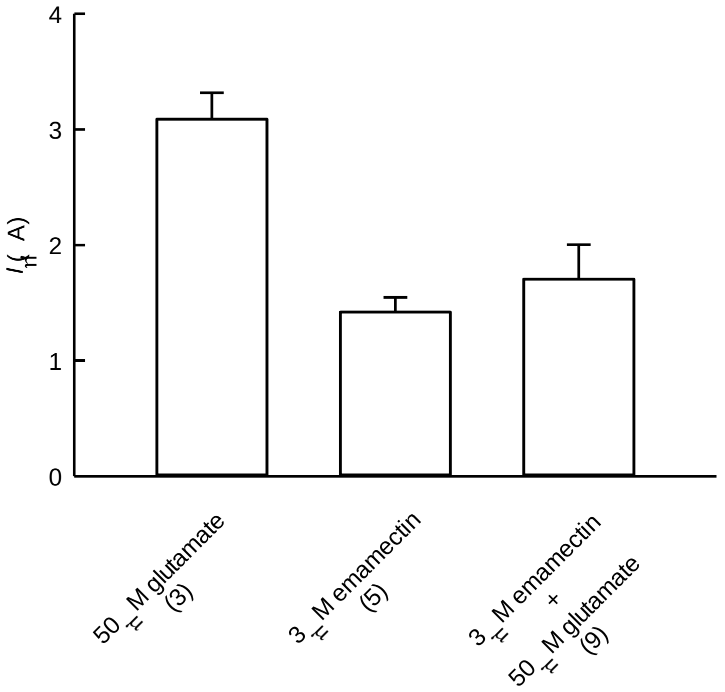

Figure S3. Comparison of the effects of saturation concentrations of glutamate and emamectin on CrGluClα-mediated current measured after expression in Xenopus oocytes. Data are means ± SEM of number of experiments given in brackets.
