# Supplementary material for: Identification and Functional Expression of a Glutamate- and Avermectin-Gated Chloride Channel from Caligus rogercresseyi, a Southern Hemisphere Sea Louse Affecting Farmed Fish
Source: PLoS Pathog. 2014 Sep 25;10(9):e1004402. doi: 10.1371/journal.ppat.1004402 (PMC4177951; doi:10.1371/journal.ppat.1004402)
Supplement: Figure S4 — Homology model of Caligus rogercresseyi glutamate receptor CrGluClα. A: Vista lateral view. B. Upper view from the extracellular side. The protein is shown in Newcartoon drawing method. Transmembrane α-helices (M1–M4) are in blue, extracelluar β-sheets are shown in red, and coils in yellow. (PDF) [file ppat.1004402.s004.pdf]

Figure S4

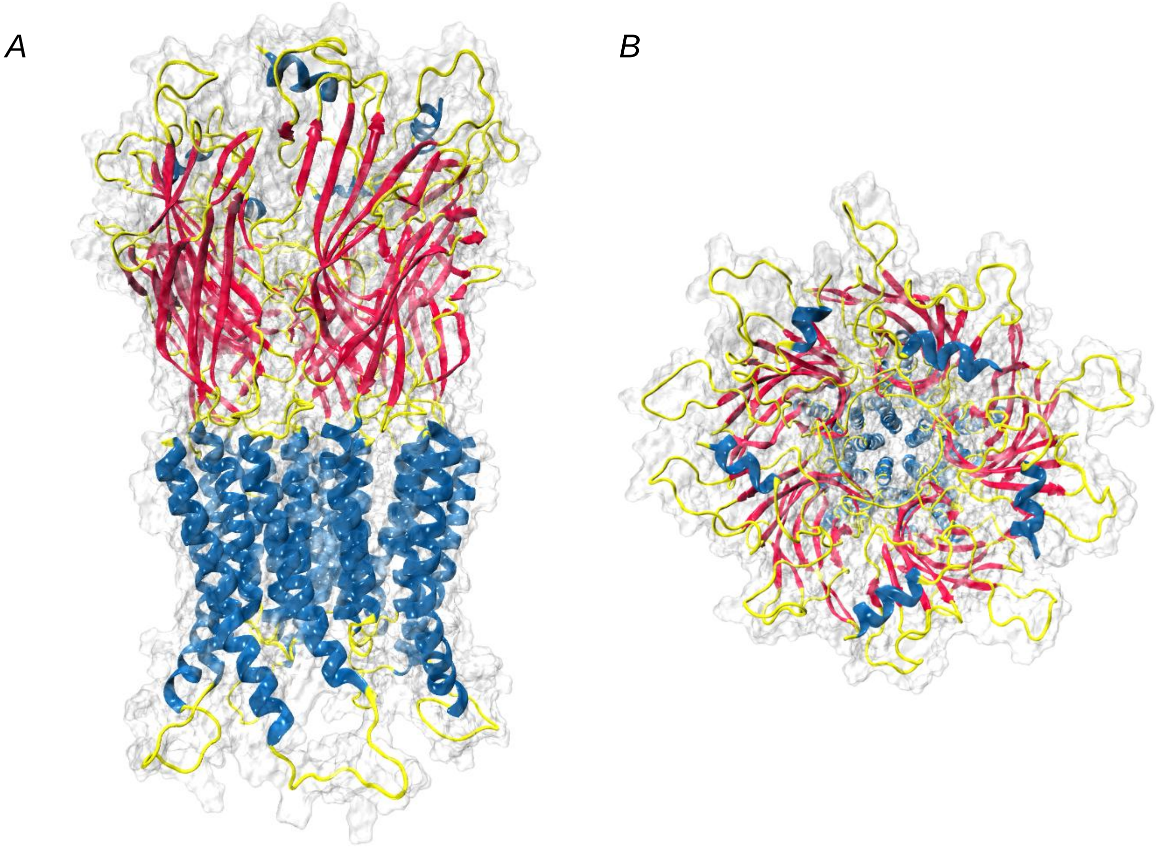


Figure S4. Homology model of *Caligus rogercresseyi* glutamate receptor CrGluCl$\alpha$. A: Vista lateral view. B. Upper view from the extracellular side. The protein is shown in Newcartoon drawing method. Transmembrane $\alpha$-helices (M1-M4) are in blue, extracelular $\beta$-sheets are shown in red, and coils in yellow.
