# Supplementary material for: Identification and Functional Expression of a Glutamate- and Avermectin-Gated Chloride Channel from Caligus rogercresseyi, a Southern Hemisphere Sea Louse Affecting Farmed Fish
Source: PLoS Pathog. 2014 Sep 25;10(9):e1004402. doi: 10.1371/journal.ppat.1004402 (PMC4177951; doi:10.1371/journal.ppat.1004402)
Supplement: Figure S5 — Chemical structures of ivermectin and emamectin. Sites involved in H-bond interactions with CrGluα as suggested by the docking assays are indicated. M2 and M3 belong to one subunit whilst M1 is in the neighbouring subunit. Images are from the PubChem (http://pubchem.ncbi.nlm.nih.gov/) database. (PDF) [file ppat.1004402.s005.pdf]

Figure S5

Ivermectin

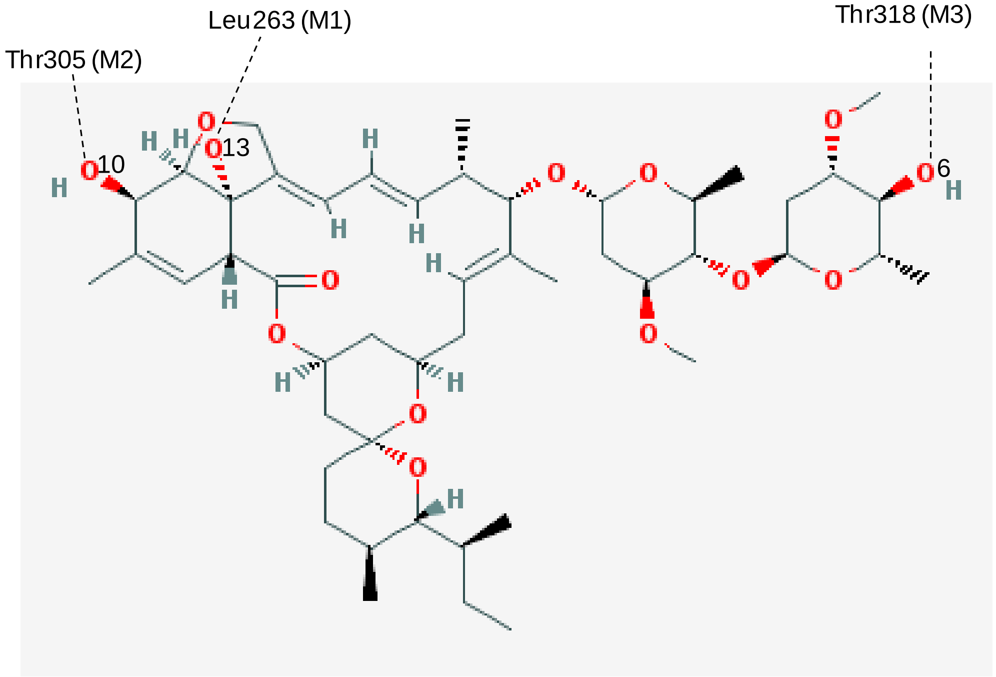

Emamectin

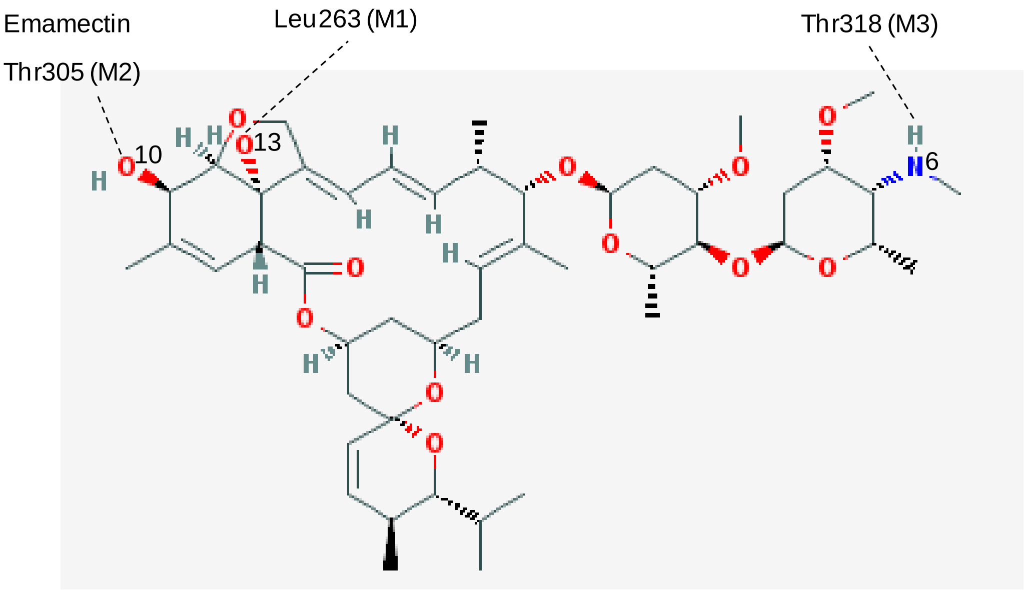

Figure S5. Chemical structures of ivermectin and emamectin. Sites involved in H-bond interactions with CrGluClα as suggested by the docking assays are indicated. M2 and M3 belong to one subunit whilst M1 is in the neighbouring subunit. Images are from the PubChem (http://pubchem.ncbi.nlm.nih.gov/) database.
