# Supplementary material for: Identification and Functional Expression of a Glutamate- and Avermectin-Gated Chloride Channel from Caligus rogercresseyi, a Southern Hemisphere Sea Louse Affecting Farmed Fish
Source: PLoS Pathog. 2014 Sep 25;10(9):e1004402. doi: 10.1371/journal.ppat.1004402 (PMC4177951; doi:10.1371/journal.ppat.1004402)
Supplement: Table S1 — Primers used in PCR reactions at various stages of the cloning of C. rogercresseyi GluClα receptor. (PDF) [file ppat.1004402.s006.pdf]

Table S1

Oligonucleotide name	Oligonucleotide sequence
cDNA Cloning Primer	5’-GGCCACGCGTCGACTAGTACT(17)V-3’
PCR RACE 3’	5’-GGCCACGCGTCGACTAGTAC-3’
T-S primer	5’-CACCATCGATGTCGACACGCGTCGGG-3’
T-S PCR	5’-CATCGATGTCGACACGCG-3’
PCRdegFor(d64)	5’-AATGGAATATTCTGTTCAACTTacnttymgnga-3’
PCRdegRev(d16)	5’-CAGCTGTACTCCCTGTrttngtytt-3’
PCRiFor1	5’-ATTGCTAGTTATGGATGGACC-3’
PCRiFor2	5’-CCTGGTATACATTTGGAAGTCC-3’
PCRiRev1	5’-AGCGTCAATGAAACCCTAATGC-3’
PCRiRev2	5’-GAATGATGTTATGGAAATGGCC-3’
PCRfullFor	5’-TGGATGAGATGGTTCTTTGTC-3’
PCRfullRev	5’-TCCAGTCCTTTATCATTTATCAAC-3’
PCRmidFor	5’-GGCCATTTCCATAACATCATTC-3’
PCRmidRev	5’-CGAGTAAGGCACTGAAGACG-3’
PCR3’For	5’-AAGAGAGTTTTCATATTACCTC-3’
PCR3’Rev	5’-ATTTATCAACTTTATTTGCCC-3’

Table S1. Primers used in PCR reactions at various stages of the cloning of *C. rogercresseyi* GluClα receptor.
