# Supplementary material for: Identification and Functional Expression of a Glutamate- and Avermectin-Gated Chloride Channel from Caligus rogercresseyi, a Southern Hemisphere Sea Louse Affecting Farmed Fish
Source: PLoS Pathog. 2014 Sep 25;10(9):e1004402. doi: 10.1371/journal.ppat.1004402 (PMC4177951; doi:10.1371/journal.ppat.1004402)
Supplement: Table S2 — Summary of amino acid differences in full length clones. The upper row shows the two possible options of amino acid or deletions with their respective positions. The bottom row shows the number of times they were found. Highlighted in grey are shown the groups that were selected as different branches to build the phylogenetic tree of Figure 2 in the main text. (PDF) [file ppat.1004402.s007.pdf]

Table S2

		FULL CLONES																	
	amino acid	12	20	22	24	27	31	48	73	80	173	225	236	269	301	347	376-377	387	411
	position	F/Y	Δ/I	I/L	C/Y	I/V	A/T	Q/K	G/D	S/Y	S/L	S/P	Y/C	P/S	T/M	P/S	Δ/AS	R/K	L/Q
1	CrGluCl-Dw1.3	F	Δ	I	C	V	A	Q	D	S	S	P	C	P	T	S	AS	K	L
2	CrGluCl-Dw1.15	F	Δ	I	C	V	A	Q	D	S	S	P	C	P	T	S	AS	K	L
3	CrGluCl-Dw1.16	F	Δ	I	C	V	A	Q	D	S	S	P	Y	P	T	S	AS	K	L
4	CrGluCl-Dw1.1	F	Δ	I	C	V	A	Q	D	S	S	P	Y	P	T	S	AS	K	L
5	CrGluCl-Vald8	F	Δ	I	C	V	A	Q	D	S	S	P	Y	P	T	S	AS	K	L
6	CrGluCl-Vald17	F	Δ	I	C	V	A	Q	D	S	S	P	Y	P	T	S	AS	K	L
7	CrGluCl-Vald13	F	Δ	I	C	V	A	Q	D	S	S	P	Y	P	T	S	AS	K	L
8	CrGluCl-Vald20	F	I	I	C	V	A	Q	D	S	S	P	Y	P	T	S	AS	K	L
9	CrGluCl-Dw1.11	F	I	I	C	V	A	Q	D	S	S	P	Y	P	T	S	AS	K	L
10	CrGluCl-Vald11	F	I	I	C	V	A	Q	D	S	S	P	Y	P	T	S	AS	R	L
11	CrGluCl-Vald15	F	I	I	C	V	A	Q	D	S	S	P	Y	P	T	S	AS	R	L
12	CrGluCl-Dw1.13	F	Δ	I	C	I	A	Q	D	S	S	P	Y	P	M	S	AS	K	L
13	CrGluCl-Vald3	F	Δ	I	C	I	A	Q	G	S	S	P	Y	P	T	S	AS	R	Q
14	CrGluCl-Vald16	F	Δ	I	C	I	A	Q	G	S	S	P	Y	P	T	S	AS	R	Q
15	CrGluCl-Err9	F	I	I	C	V	A	Q	G	S	S	P	Y	P	T	S	AS	R	Q
16	CrGluCl-Dw1.2	F	Δ	I	Y	V	A	Q	D	S	S	P	Y	P	T	S	AS	R	Q
17	CrGluCl-Dw1.14	Y	I	I	C	V	A	K	D	S	S	P	Y	P	T	S	AS	R	Q
18	CrGluCl-Vald7	F	I	I	C	V	A	Q	D	S	L	P	Y	S	T	S	AS	R	Q
19	CrGluCl-Dw1.6	F	Δ	I	C	V	A	Q	D	Y	S	P	Y	P	T	P	AS	R	Q
20	CrGluCl-Vald2	F	Δ	I	C	I	A	Q	G	S	S	P	Y	P	T	S	Δ	R	Q
21	CrGluCl-Err1	F	Δ	I	C	V	A	Q	D	S	S	P	Y	P	T	S	Δ	R	Q
22	CrGluCl-Err4	F	I	I	C	V	A	Q	G	S	S	P	Y	P	T	S	Δ	R	L
23	CrGluCl-Err5	F	I	I	C	V	A	Q	G	S	S	P	Y	P	T	S	Δ	R	L
24	CrGluCl-Vald10	F	I	I	C	V	A	Q	D	S	S	P	Y	P	T	S	Δ	R	L
25	CrGluCl-Vald14	F	Δ	I	C	V	A	Q	D	S	S	P	Y	P	T	S	Δ	K	L
26	CrGluCl-Vald19	F	I	I	C	V	A	Q	D	S	S	S	Y	P	T	S	Δ	K	L
27	CrGluCl-Err10	F	Δ	L	C	V	A	Q	G	S	S	P	Y	P	T	S	Δ	K	L
28	CrGluCl-Dw2.4	F	I	I	C	V	A	Q	G	S	S	P	Y	P	T	S	AS	R	L
29	CrGluCl-Dw2.6	F	I	I	C	V	A	Q	G	S	S	P	Y	P	T	S	AS	R	L
30	CrGluCl-Dw2.7	F	I	I	C	V	A	Q	G	S	S	P	Y	P	T	S	AS	R	L
31	CrGluCl-Dw2.5	F	I	I	C	V	T	Q	D	S	S	P	Y	P	T	S	AS	R	Q
32	CrGluCl-Dw2.8	F	I	I	C	V	A	Q	D	S	S	P	Y	P	T	S	AS	R	Q
33	CrGluCl-Dw1.7	F	Δ	I	C	V	A	Q	D	S	S	P	Y	P	T	S	AS	K	L
		32/1	17/16	32/1	32/1	4/29	32/1	32/1	10/23	32/1	32/1	32/1	31/2	32/1	32/1	32/1	8/25	19/14	22/11

Table S2. Summary of amino acid differences in full length clones. The upper row shows the two possible options of amino acid or deletions with their respective positions. The bottom row shows the number of times they were found. Highlighted in grey are shown the groups that were selected as different branches to build the phylogenetic tree of Figure 2 in the main text.
