# Supplementary material for: Identification and Functional Expression of a Glutamate- and Avermectin-Gated Chloride Channel from Caligus rogercresseyi, a Southern Hemisphere Sea Louse Affecting Farmed Fish
Source: PLoS Pathog. 2014 Sep 25;10(9):e1004402. doi: 10.1371/journal.ppat.1004402 (PMC4177951; doi:10.1371/journal.ppat.1004402)
Supplement: Table S3 — Summary of amino acid differences in partial clones. The differences, positions and number of times they occurred in the study are shown as for Table S2. 31, 29 and 34 clones were analyzed respectively for 5′, middle and 3′ fragments. (PDF) [file ppat.1004402.s008.pdf]

Table S3

PARTIAL CLONES																		
amino acid	12	20	22	24	27	31	48	73	80	173	225	236	269	301	347	376-377	387	411
position	F/Y	Δ/I	I/L	C/Y	V/I/A	A/T	Q/K	D/G	S/Y	S/L	P/S	Y/C	P/S	T/M	S/P	AS/Δ	K/R	L/Q
5′ fragment	31/0	20/11	31/0	29/2	27/3/1	26/5	31/0	27/4	31/0									
middle fragment										29/0	29/0	29/0						
3' fragment													29/0	34/0	34/0	22/12	20/14	22/12
	31 clones									29 clones			34 clones					

Table S3. Summary of amino acid differences in partial clones. The differences, positions and number of times they occurred in the study are shown as for Table S2. 31, 29 and 34 clones were analyzed respectively for 5’, middle and 3’ fragments.
